# Fast Alkaline Hydrothermal Synthesis of Pyrophosphate BaCr_2_(P_2_O_7_)_2_ Nanoparticles and Their NIR Spectral Reflectance

**DOI:** 10.3390/nano15130982

**Published:** 2025-06-25

**Authors:** Diego Emiliano Carrillo-Ramírez, Juan Carlos Rendón-Angeles, Zully Matamoros-Veloza, Jorge López-Cuevas, Isaías Juárez-Ramírez, Tadaharu Ueda

**Affiliations:** 1 Center for Research and Advanced Studies of National Polytechnic Institute, Saltillo-Campus, Ramos Arizpe, Coahuila 25900, Mexico; diego.carrillo@cinvestav.mx (D.E.C.-R.); jorge.lopez@cinvestav.edu.mx (J.L.-C.); 2 Graduate Division, Tecnológico Nacional de México (I.T Saltillo), Technological Institute of Saltillo, Saltillo 25280, Mexico; zully.mv@saltillo.technm.mx; 3 Ecomaterials and Energy Department, Civil Engineering Faculty, Universidad Autónoma de Nuevo León, San Nicolás de Los Garza, Nuevo León 66455, Mexico; isaias.juarezrm@uanl.edu.mx; 4 Department of Marine Resources Science, Faculty of Agriculture and Marine Science, Marine Core Research Institute, MEDi Centre, Kochi University, Nankoku 783-8502, Japan; chuji@kochi-u.ac.jp

**Keywords:** BaCr_2_(P_2_O_7_)_2_, hydrothermal synthesis, reductant agent, nanoparticles, alkaline media

## Abstract

Recently, the development of nanoparticle pigments has attracted interest in chemical preparation due to their potential functional properties, such as phosphate-based pigments. The present research focuses on the feasibility of synthesising the BaCr_2_(P_2_O_7_)_2_ pigment under hydrothermal conditions. The effect of the microstructural features of ceramic pigments (the crystalline structure, morphology, and particle size) on their optical properties (colour and reflectance) was also studied. The BaCr_2_(P_2_O_7_)_2_ compound was prepared in different fluid media, including water and NaOH solutions (0.5–1.0 M), at several reaction temperatures (170–240 °C) and intervals (6–48 h). The single-phase BaCr_2_(P_2_O_7_)_2_ did not crystallise without by-products (BaCr_2_O_10_, BaCr_2_(PO_7_)_2_) in water and the alkaline solutions, even at 240 °C for 48 h; in these fluids, the ionic Cr^3+^ species oxidised to Cr^6+^. In contrast, the BaCr_2_(P_2_O_7_)_2_ single-phase crystallisation was favoured by adding urea as a reductant agent (25.0–300.0 mmol). Monodispersed BaCr_2_(P_2_O_7_)_2_ fine particles with a mean size of 44.0 nm were synthesised at a low temperature of 170 °C for 6 h with 0.5 M NaOH solution in the presence of 50.0 mmol urea. The phosphate pigment particle grew to approximately 62.0 nm by increasing the treatment temperature to 240 °C. A secondary dissolution–recrystallisation achieved after 24 h triggered a change in the particle morphology coupled with the incrementation of the concentration of NaOH in the solution. The pyrophosphate BaCr_2_(P_2_O_7_)_2_ pigments prepared in this study belong to the green colour spectral space according to the CIELab coordinates measurement, and exhibit 67.5% high near-infrared (NIR) solar reflectance.

## 1. Introduction

Extensive attention has been paid to the preparation of inorganic pigments based on anhydrous pyrophosphates, i.e., M_2_P_2_O_7_, CaM_3_(P_2_O_7_)_2_ and M_2_(P_2_O_7_)_2_ (M = Co, Ni, among others), since they exhibit interesting physical and chemical functional properties. In addition, these pigments have promising potential in a wide variety of applications in areas including catalysis, magnetic ferroelectrics, optical sensors and luminescence [1,2,3,4,5,6,7,8,9]. In the last decade, especially new ceramic pigments in the pyrophosphate group, with various colours and high chemical stability, have attracted much attention in the chemical industry. According to the crystalline structure, the M site in the phosphate network structure can be occupied by various transition metal cations, producing a variety of solid solutions with good crystalline structural stability. In particular, Zn_2_P_2_O_7_ [10], BaCuP_2_O_7_ [11], (Mg/Cu)_2_P_2_O_7_ [12], and Ni_2_P_2_O_7_ [13] have been widely studied. The employment of chromium improves both thermal and chemical stability at elevated temperatures, which hinders the corrosion of oxide pigments by a glassy liquid phase at elevated temperatures, consequently producing satisfactory green optical tonalities in the coated material. Oxide-based nano-pigments containing chromium (III) [14], such as the spinel-structured cobalt chromium (CoCr_2_O_4_) [6,15] and cubic garnet known as Victoria green pigment (Ca_3_Cr_2_Si_3_O_12_) [6], exhibit outstanding stability. Therefore, the pyrophosphate compounds are likely candidates for obtaining a new green hue pigment incorporating Cr^3+^. Indeed, the first attempt to produce the single-phase green hue BaCr_2_(P_2_O_7_)_2_ was recently prepared via solid-state reaction; the raw reagent grade BaCO_3_, Cr_2_O_3_ and NH_4_H_2_PO_4_ powders were heated at 1200 °C for 8 h under a reducing atmosphere (H_2_:N_2_ = 5:95). In addition to its green colour tonality, the BaCr_2_(P_2_O_7_)_2_ particles with a mean particle size of 3.0 mm exhibited a high diffuse reflectance response of 85.0% in the near-infrared (NIR) spectrum between 700 and 1800 nm. A detailed crystalline structural analysis led to the establishment of the methodology for developing these oxides. However, a detailed correlation between the microstructural features (morphology and particle size) and the optical features was not found [1].

The hydrothermal processing technique has been effective in the processing of ternary inorganic pigment compounds such as the silicates (BaCuSiO_6_), which can be crystallised with controlled 3D hierarchical architectures produced by nanostructured primary crystals, followed by spontaneous self-assembly during the growth of the fine pigment aggregates [16]. Our group has recently investigated the synthesis of the analogous silicate pigment Victorian green containing Cr^3+^ ions under alkaline hydrothermal conditions. The crystallisation of the Ca_3_Cr_2_Si_3_O_12_ proceeded in highly concentrated NaOH solutions between 2.5–5.0 M at 200 °C for 3 h via a single-step chemical reaction. The garnet-structured particles synthesised by the dissolution of a precursor Ca_3_Cr_2_Si_3_(OH)_24_ coprecipitated; the solute crystallised simultaneously to form euhedral-shaped nanometric-sized crystals (approximately 10 nm) that self-assembled to form 3D hierarchical popcorn-shaped Ca_3_Cr_2_Si_3_O_12_ aggregates with sizes in the range of 66.0–156.0 nm [17]. Despite the severe conditions, the oxidation of the precursor Cr^3+^ ions did not occur in the highly concentrated alkaline hydrothermal medium without reductant reagents and particle-size organic modifiers to assist the crystallisation process. Additionally, the optical diffuse reflectance measurements in the NIR (700–2500 nm) revealed that the hydrothermally prepared Victorian green aggregates exhibit a high response of 95.0%, proving this compound’s potential as a cold pigment [18].

The single-phase BaCr_2_(P_2_O_7_)_2_ has not been prepared before by a soft chemical processing route, i.e., hydrothermal processing, to the best of our knowledge. Therefore, in this study, we have evaluated the feasibility of obtaining the BaCr_2_(P_2_O_7_)_2_ crystalline phase via a single-step reaction in alkaline solutions under hydrothermal conditions, and simultaneously, controlling the microstructural aspect (size and morphology) of the particles towards optimising the NIR solar reflectance and the colour chromatic properties of this green pigment. In addition, a systematic analysis devoted to reducing the chemical oxidation potential of the alkaline media was carried out by adding a reductant agent (urea), due to the oxidation reaction of Cr^3+^ to Cr^6+^ being the major chemical challenge that might hinder the single-step reaction, to solely produce the target BaCr_2_(P_2_O_7_)_2_ phase. Hence, the preliminary experiments aimed to produce the pyrophosphate pigment in mildly acidic and concentrated NaOH (0.5–1.0 M) solutions under hydrothermal conditions from an ecological point of view. In addition, the use of urea reactant prevents the oxidation of Cr^3+^ to Cr^6+^, hindering the presence of Cr(VI)-containing species as by-products. UV-vis NIR structural analyses of BaCr_2_(P_2_O_7_)_2_ green pigments were carried out to discuss the differences in the optical pigment properties based on the particle size variation caused by the variations in the experimental parameters (temperature and reaction interval).

## 2. Materials and Methods

### 2.1. Materials

The pyrophosphate BaCr_2_(P_2_O_7_)_2_ particles were prepared using chemical-grade reagents of barium nitrate [Ba(NO_3_)_2_] (Sigma-Aldrich, Milwaukee, Wisconsin, USA, 99.0% purity), chromium nitrate nanohydrate [Cr(NO_3_)_3_·9H_2_O] (Sigma-Aldrich, Milwaukee, WI, USA, 99.0% purity) and phosphoric acid [H_3_PO_4_] (Sigma-Aldrich, Milwaukee, WI, USA, 85.0% purity), which were used for the precursor gel coprecipitation. The precursor solutions were prepared with deionised water (Hycel, Monterrey, N.L., Mexico) with various concentrations of 0.05 M Ba(NO_3_)_2_, 0.1 M Cr(NO_3_)_3_ and 0.2 M H_3_PO_4_. The NaOH (Sigma-Aldrich, Milwaukee, WI, USA, 99.0% purity) solution (0.5–1.0 M) was used as a hydrothermal solvent to control the solution pH, and urea powder reagent grade [Sigma-Aldrich Milwaukee, WI, USA, 99.0% purity, CO(NH_2_)_2_] was a reducing agent to prevent the oxidation of Cr^3+^ to Cr^6+^. TiO_2_ rutile-structured powder (Sigma-Aldrich 99.0% purity, <5.0 μm) was selected as a standard for comparison of the pyrophosphate optical properties characterisation.

### 2.2. Hydrothermal Treatments

Reaction mother liquor for the hydrothermal treatments was prepared by mixing equal volumes of the precursor solutions, with a Ba:Cr:P ratio of 7.5:7.5:7.5 mL, which corresponds to the pyrophosphate stoichiometric cationic Ba:Cr:P molar ratio of 1:2:4, adding Ba, Cr and P-containing solutions into the Teflon chamber bottom; and pouring 12.5 mL of water or NaOH solution. In a different set of experiments, urea powder [CO(NH_2_)_2_, 25 to 300 mmol/dm^3^] was placed at the bottom of the autoclave before adding any solution. It is noted that no precipitation occurred from the mixture liquor when water was mixed in, whilst a milky green colloid spontaneously coprecipitated on the addition of the NaOH solution. All experiments were carried out at a constant mother liquor solution volume of 35 mL, corresponding to a 50% autoclave filling ratio. The preliminary hydrothermal treatments were carried out at different reaction intervals (6–48 h) and various temperatures (170–240 °C) to investigate the chemical equilibrium that produces solely BaCr_2_(P_2_O_7_)_2_. After each treatment, the reaction products were vigorously washed three times with hot water (80 °C) to clean off the remaining mother liquor; the resultant powders were then dried overnight in an oven at 70 °C.

### 2.3. Characterisation

Powder X-ray diffraction (XRD): The analyses were conducted in an X-ray diffraction apparatus (Empyrean, Malvern Panalytical, Almelo, The Netherlands). Each sample was scanned at the following conditions: 40 kV and 20 mA, using Cu-K_a_ radiation (λ = 1.5418 Å). The XRD patterns were recorded in the 2θ range of 10–90° at a constant scanning speed of 10°/min with a step of 0.02°. Rietveld refinement analysis was carried out using the TOPAS 4.2 (Bruker AXS: Karlsruhe, Germany, 2009) software. The space group and the atomic spatial positions (Wyckoff number and coordinates) of the BaCr_2_(P_2_O_7_)_2_ crystalline structure were determined based on the card MP-1192170, German crystallographic database [19], shown in Table 1. Details of the parameters considered in the refining algorithm and the secondary phase crystallographic information (Table S1) are described in Section S1 of the Supplementary Supporting Information File (hereafter referred to as SSIF).

X-ray photoelectron spectroscopy (XPS): Compositional analyses of selected pigment powder samples prepared with and without urea were conducted by XPS. All the analyses were conducted by selecting the carbonaceous C 1s peak (284.6 eV) as a standard calibrating binding energy. The spectra were recorded on a Thermo Fisher Scientific Model K-Alpha, Waltham, MA, USA.

Fourier transform infrared spectroscopy (FT-IR): Additional structural details associated with atomic bond differences in the BaCr_2_(P_2_O_7_)_2_ and the presence of water molecules were investigated by FT–IR analyses, which were conducted using JASCO 4000 equipment (Hachioji, Tokyo, Japan). Pelletised samples consisting of 5 mg pigment powder and 200 mg KBr were used for the analyses. The powder samples were dried overnight at 60 °C before pellet preparation.

Morphology and microstructural observations: The microstructural aspects of the pyrophosphate BaCr_2_(P_2_O_7_)_2_ particles were observed using a field emission scanning electron microscope (FE-SEM JEOL JSM–7100F, Tokyo, Japan). The microscope was operated at 10 kV and 69 mA. The statistical analysis of particle size and its distribution was calculated using SEM images, considering 80 particles. Crystalline structural details of pyrophosphate particles were revealed by high-resolution transmission electron microscopy (HR–TEM, Philips Talos F200X, Seoul, Republic of Korea) operated at 200 kV.

Differential scanning calorimetry (DSC). The thermal stability of BaCr_2_(P_2_O_7_)_2_ powders was evaluated via thermogravimetry (TG) and differential scanning calorimetry analysis (Mettler Toledo 3+ DTA/DSC, Los Angeles, CA, USA). The sample was heated from 30 to 900 °C at a constant heating rate of 10 °C/min in an air atmosphere.

Optical properties. The colour CIELab* and reflectance spectra of pyrophosphate BaCr_2_(P_2_O_7_)_2_ powder were measured in a UV-Visible–NIR spectrometer (Jasco V-770, Hachioji, Tokyo, Japan) equipped with an integrating sphere device. Baseline calibration was carried out with BaSO_4_ fine powder as the reference, and the spectrometer colour space parameters were measured according to the standard CIELab* colourimetry method. Additional details on the measurement of band-gap, the chromatic parameters and the solar reflectance of the pigments are described in detail in Section SF1 in the SSIF.

## 3. Results and Discussion

The one-pot hydrothermal processing treatment was employed as a new chemical route for preparing synthetic BaCr_2_(P_2_O_7_)_2_ pigment particles, which have been prepared solely by the conventional solid-state reaction at high temperatures (1200 °C). Hence, the appropriate reaction conditions were investigated in terms of the solution pH to trigger the crystallisation of only the single-phase BaCr_2_(P_2_O_7_)_2_, taking into consideration the prevention of the oxidation of the precursor Cr^3+^ to Cr^6+^ by addition of a reductant agent (urea, CO(NH_2_)_2_) under hydrothermal conditions [20]. Table 2 summarises the relevant reaction conditions of selected experiments and the structural information on the reaction products calculated by Rietveld Analysis. Further details of all the experiments conducted in this work are given in Table S1 of the SSIF. The condition of the mother liquor strongly depends on the particle crystallisation under the hydrothermal treatment. In the case of water (pH 6.2), the formation of an amorphous powder is triggered due to the low chemical stability of the mother liquor under hydrothermal conditions. A different reaction pattern was observed in the case of the alkaline solutions, but two different crystalline phases, including the BaCr_2_(P_2_O_7_)_2_ pigment, were obtained in these experiments, as discussed in the next section.

### 3.1. Chemical Stability of the Pyrophosphate BaCr_2_(P_2_O_7_)_2_ Single-Phase Under Acidic and Alkaline Hydrothermal Conditions

**Figure 1:**
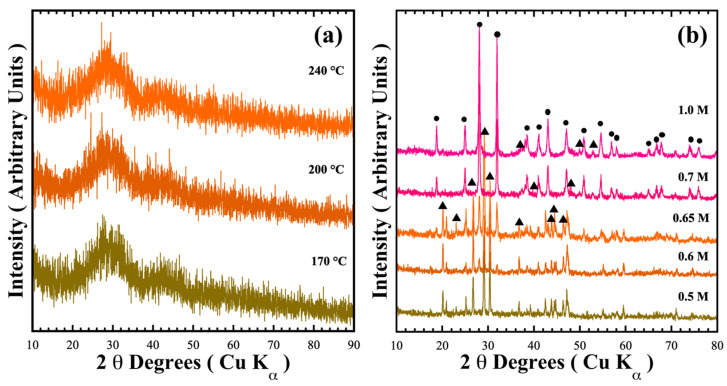
XRD patterns of the reaction products obtained under hydrothermal conditions for 48 h with (**a**) water varying the temperature, and (**b**) at 240 °C with different NaOH solution concentrations. Crystalline phases: (●) BaCr_2_(P_2_O_7_)_2_ and (▲) BaHCr_2_PO_10_.

Figure 1 shows the typical X-ray diffraction patterns of reaction products hydrothermally obtained at mildly acidic (pH = 6.17) and mild alkaline (pH = 12.5–13.59) conditions. In mildly acidic conditions, a green powder formed in the mother liquor irrespective of the treatment temperature; which exhibited no trace of crystalline phases, instead showing the amorphous phase (Figure 1a). Although precipitation proceeds in the mildly acidic media with counter NO_3_^−^ anions, we surmise that crystallisation could not be triggered because the solute supersaturation stage to produce the metastable state required for embryo crystallisation is not reached in the fluid.

On the other hand, the treatment conducted in NaOH alkaline media provoked a different reaction pathway to form two major crystalline phases (Figure 1b). The peaks of the crystalline phases obtained in the 0.5 M NaOH solution were indexed with those of the pyrophosphate compounds BaCr_2_(P_2_O_7_)_2_ (●, MP Card No. 1192170) and BaHCr_2_PO_10_ (▲, COD card no. 96-901-6408). The content of each phase indexed on each XRD powder pattern was determined by a computer algorithm, which comprises two subroutines containing the crystallographic phase (spatial coordinates and Wykoff values) shown in Table 1 and Table S1 in the SSIF. Figure S2a,b show examples of the refinement calculation. The accuracy of the phase content computation is supported by the low values of the GOF χ^2^ parameter, which is associated with the algorithm’s refinement goodness. The ratio of single-phase BaCr_2_(P_2_O_7_)_2_ increased with the gradual augmentation of the NaOH concentration; the maximum yield of BaCr_2_(P_2_O_7_)_2_ was 75.9 ± 0.8 wt.% in the 0.65 M NaOH solution. In the 1.0 M NaOH solution, the target pyrophosphate pigment amount remained almost constant (74.0 ± 1.8 wt.%). These results indicate that the precursor coprecipitated a hydrous gel identified by Equation (1) (BaCr_2_P_4_(OH)_28_∙xH_2_O_gel_) rapidly reacted in the alkaline media during the hydrothermal treatment. The chemical equilibrium that triggers the crystallisation of pyrophosphate compounds should be Equation (2), through the dimerisation of phosphate into pyrophosphate achieved in the pH alkaline range of 12.61–13.61 [21]. However, the coexistence of NO_3_^−^ in the mother liquor could oxidise Cr^3+^ into Cr^6+^, resulting in the prevention of the complete crystallisation of the precursor hydrated gel BaCr_2_P_4_(OH)_28_∙xH_2_O to BaCr_2_(P_2_O_7_)_2_ pigment. To overcome this issue, addition of a reductant agent, namely urea, to prevent chromium oxidation, was used to prepare the sole formation of BaCr_2_(P_2_O_7_)_2_ [20].

(1) $$Ba{\left({NO}_{3}\right)}_{2(aq)}+2Cr{\left({NO}_{3}\right)}_{3(aq)}+4H_{3}PO_{4(aq)}\overset{NaOH_{\left(aq\right)}}{\to }\;Ba{Cr}_{2}P_{4}{\left(OH\right)}_{28}\cdot xH_{2}O_{\left(gel\right)}+8{NO}_{3(aq)}^{-}+4H_{2}O+{Na}_{\left(aq\right)}^{+}$$

(2) $$Ba{Cr}_{2}P_{4}{\left(OH\right)}_{28}\cdot xH_{2}O_{\left(gel\right)}+8{NO}_{3(aq)}^{-}+4H_{2}O+{Na}_{\left(aq\right)}^{+}\overset{∆T}{\to }\;xBa{Cr}_{2}{\left(P_{2}O_{7}\right)}_{2(s)}+yBaH{Cr}_{2}PO_{10(s)}+8{NO}_{3(aq)}^{-}+4H_{2}O+{Na}_{\left(aq\right)}^{+}$$

The new series of preparation were conducted specifically at 240 °C for 48 h in a 0.7 M NaOH alkaline solution in the presence of designated concentrations of urea (25.0–300.0 mmol/dm^3^) under hydrothermal conditions to form green powders. Although the pigment synthesis proceeded at mild alkaline conditions of pH 9.46–10.66, the polyatomic anions (HCO_3_^−^) generated from urea decomposition decreased the pH of the mother liquor, which is likely to promote a reductive environment in mildly alkaline media.

**Figure 2:**
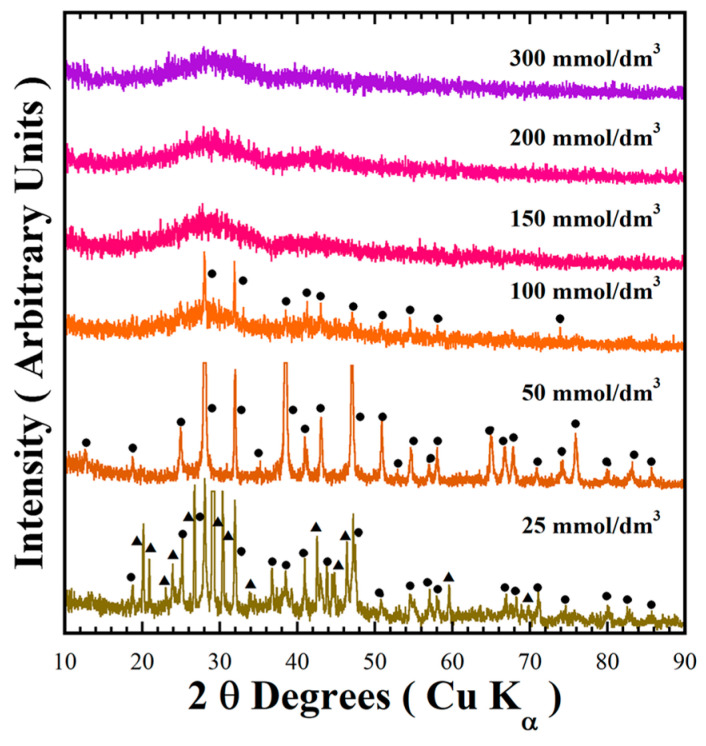
XRD patterns of the reaction products obtained at 240 °C for 48 h using a 0.7 M NaOH solution with different urea contents. Crystalline phases (●) BaCr_2_(P_2_O_7_)_2_ and (▲) BaHCr_2_PO_10_.

Figure 2 shows the typical XRD pattern of the green powders obtained via the standard conditions mentioned above, with variation of the urea concentration. In the presence of 25 mmol/dm^3^ of urea, two phases of BaCr_2_(P_2_O_7_)_2_ and BaHCr_2_PO_10_ formed simultaneously. At the same time, the single-phase BaCr_2_(P_2_O_7_)_2_, for which the peaks in the XRD pattern were indexed with those in the MP Card No. 1192170, was formed in the presence of 50 mmol/dm^3^ urea, which is appropriate for completion of the precursor gel dissolution. Subsequently, solvent supersaturation with the solute triggers BaCr_2_(P_2_O_7_)_2_ embryonic nucleation and continuous growth, as suggested by the single-step chemical reaction given in Equation (3). The addition of 100 mmol/dm^3^ urea contents resulted in the formation of two phases: the primary BaCr_2_(P_2_O_7_)_2_ and amorphous species (Figure 2). The amount of the primary BaCr_2_(P_2_O_7_)_2_ phase gradually decreased with increasing amounts of urea (see Table 2). In the presence of >150 mmol/dm^3^ urea, the amorphous powder was formed during treatments conducted for 48 h. Interestingly, this reaction pathway is analogous to that observed under mildly acidic conditions. Urea saturation decreased the solvent pH from 12.88 to 9.7 (see Table 2), leading to limited pyrophosphate crystallisation because the solute supersaturation condition for triggering crystallisation did not occur at lower pH. Hence, the BaCr_2_(P_2_O_7_)_2_ particles crystallisation occurred preferentially at a mild pH range of 10.16 to 10.52 by a single-step reaction involving a dissolution–crystallisation mechanism [18]. The urea reductant agent controls the reaction media pH and avoids chromium (III) oxidation. Under these conditions, the precursor gel dehydration and dissolution occurred in the alkaline buffer medium. Consequently, solute supersaturation was reached in the solvent media, triggering the BaCr_2_(P_2_O_7_)_2_ compound crystallisation without byproducts, according to Equation (3).

(3) $$Ba{Cr}_{2}P_{4}{\left(OH\right)}_{28}∙xH_{2}O_{\left(gel\right)}+8{NO}_{3(aq)}^{-}+4H_{2}O+2NH_{3(aq)}^{-}+HCO_{3(aq)}^{-}\overset{NaOH_{\left(aq\right)}}{\to }\;Ba{Cr}_{2}{\left(P_{2}O_{7}\right)}_{2(s)}+8{NO}_{3(aq)}^{-}+4H_{2}O+2{NH}_{3(aq)}^{-}+{Na}_{\left(aq\right)}^{+}+H{CO}_{3(aq)}^{-}$$

### 3.2. Tailoring the BaCr_2_(P_2_O_7_)_2_ Pigment Synthesis Assisted by Urea Under Hydrothermal Conditions

**Figure 3:**
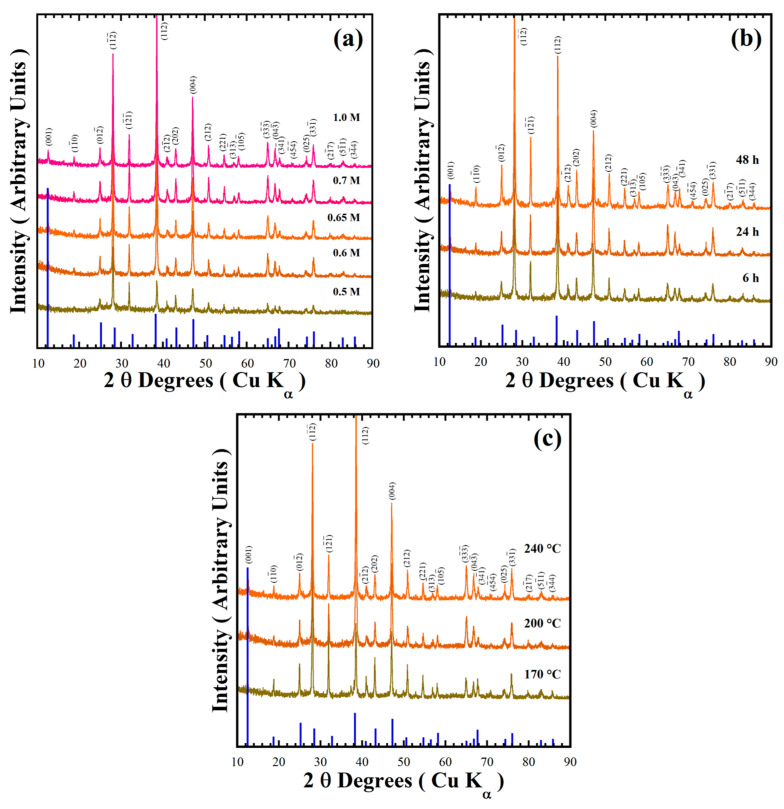
X-ray diffraction patterns of BaCr_2_(P_2_O_7_)_2_ powders prepared with a concentration of 50 mmol/dm^3^ of urea, at 240 °C for 24 h varying (**a**) the NaOH solution concentration; (**b**) with a 0.7 M NaOH solution for different reaction times; and (**c**) for 24 h with a 0.7 M NaOH solution at various reaction temperatures. The blue solid lines correspond to the BaCr_2_(P_2_O_7_)_2_ single XRD pattern.

Figure 3 shows XRD patterns of the powder products prepared in various conditions of mother liquors containing designated concentrations of NaOH (0.5–1.0 M) in the presence of 50 mmol/dm^3^ urea at different reaction temperatures (170–240 °C) for various reaction times (6–48 h) to check the stability of the pyrophosphate compounds. The major peak of the triclinic structured BaCr_2_(P_2_O_7_)_2_ with Miller index (001), observed at a 2θ angle of 12.50°, is too small in intensity [19]. This peak intensity did not increase with increasing NaOH concentration, while a gradual increase in intensity of the peak at a 2θ angle of 38.22° (as seen in Figure 3a) implied the preferential crystal growth of the BaCr_2_(P_2_O_7_)_2_ particles along the [112] crystallographic direction. The BaCr_2_(P_2_O_7_)_2_ single-phase crystallisation proceeds in mildly basic media; the urea decomposed above 120 °C to keep the Cr^3+^ species stable (Equation (3)).

All diffraction patterns of the powders prepared in a 0.7 M NaOH solution at 240 °C for different reaction times were indexed with those of the BaCr_2_(P_2_O_7_)_2_ compound (Figure 3b). The reaction time did not remarkably affect the stability of the pyrophosphate pigment, indicating a short reaction time of 6 h is enough to obtain the single phase BaCr_2_(P_2_O_7_)_2_ crystal. Below a reaction interval of 24 h, the particles preferentially grew parallel to the crystallographic plane with Miller indexes (112) at 2θ = 38.22°. However, the particles coarsened above 24 h reaction time, and the peak at 2θ = 28.1°, parallel to the Miller index plane (1$\bar{1}\bar{2}$), which belongs to the {112} plane family, for the powder prepared for 48 h, increased remarkably in intensity (Figure S1 in the SSIF). A dissolution–recrystallisation reaction should occur, resulting in the change in particle coarsening associated with the increase in diffraction peaks intensities due to local volume molar changes of the solvent under hydrothermal conditions. The single-phase BaCr_2_(P_2_O_7_)_2_ compound was obtained even at 170 °C for 24 h (Figure 3c). Finally, the single-phase BaCr_2_(P_2_O_7_)_2_ powder was prepared in the NaOH solution at a sharp pH range of 10.16–10.53 in presence of 50.0 mmol/dm^3^ under hydrothermal conditions at 170–200 °C for less than 6 h.

**Figure 4:**
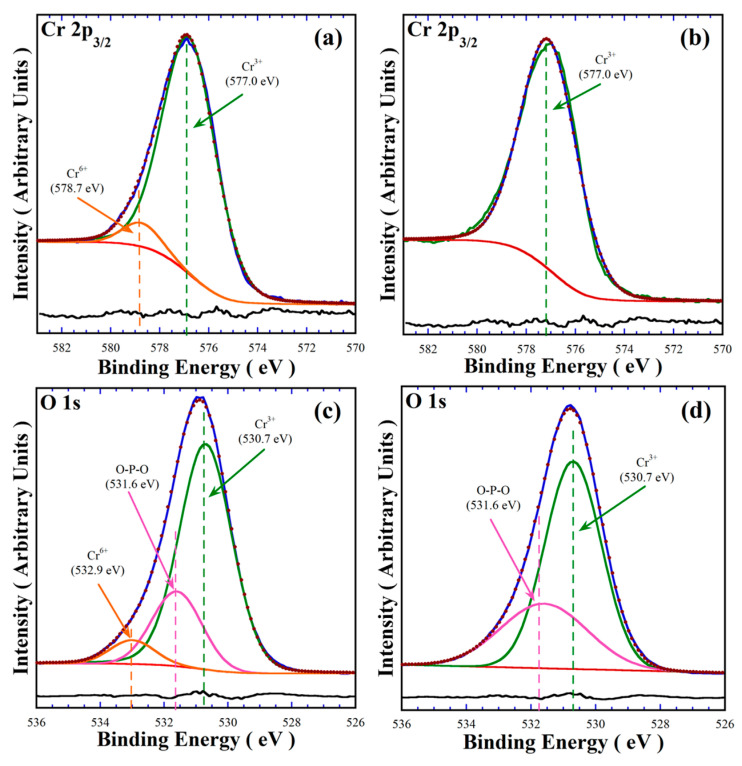
XPS spectra of pigment powders prepared (**a**,**c**) in the absence of urea, and (**b**,**d**) with the addition of 50 mmol/dm^3^ of urea. The green dashed line corresponds to the Cr^3+^ oxidation state, the orange line represents the Cr^6+^ species, and the pink line indicates the O-P-O bridging group associated with the pyrophosphate structure.

The XRD results indicate that the oxidation reaction of Cr^3+^ to Cr^6+^ partially occurs in the hydrothermal fluid without addition of the urea agent. XPS analyses of the powders prepared at standard reaction conditions at 240 °C for 48 h in 0.7 M NaOH, in the absence and in the presence of urea as a reductant agent, were conducted to determine the effect of urea addition in terms of limitation of the oxidation reaction of Cr^3+^ to Cr^6+^ in the hydrothermal system. The typical XPS spectra of the Cr 2p_3/2_ and O 1s core levels are shown in Figure 4. The deconvolution of the Cr 2p_3/2_ spectrum of the powders synthesised in the absence of urea showed that this peak with a FHWM of 2.754 eV is constituted by two Cr oxidation states that correspond to the signals at energy binding levels of 577.0 eV and 578.7 eV, which correspond to Cr^3+^ and Cr^6+^ species (Figure 4a), respectively [22,23]. In contrast, the deconvolution of the core level Cr 2p_3/2_ spectrum for the powders prepared in the solution containing 50.0 mmol urea only proceeded by the binding energy at 577.0 eV, which corresponds to the Cr^3+^ cation (Figure 4b), because the FHWM (2.686 eV) of the deconvoluted peak matched the same position in the XPS experimental peak. The linearity of the residual line calculated by subtracting the deconvoluted spectrum from the experimental one supports that adding urea can maintain the valence of Cr^3+^ in alkaline aqueous media. Furthermore, the analyses of the O 1s core-level of the samples prepared without (Figure 4c) exhibited three different peaks at the binding energies of 530.7 eV, 531.6 eV and 532.9 eV, corresponding to the oxygen coordinated to Cr^3+^, the O-P-O oxygen bridging in the pyrophosphate group, and to Cr^6+^-O, respectively, while the spectrum of the sample prepared in the presence of urea only comprises two binding energy peaks at 530.7 eV and 531.6 eV of the oxygen binding O-P-O in the P_2_O_7_ units and the oxygen binding to Cr^3+^ species, respectively (Figure 4d). These results are quantitatively consistent based on the peak areas and the residual line shapes portrayed in Figure 4c,d.

### 3.3. Structural Features of the BaCr_2_(P_2_O_7_)_2_ Powders Prepared Under Hydrothermal Conditions

**Figure 5:**
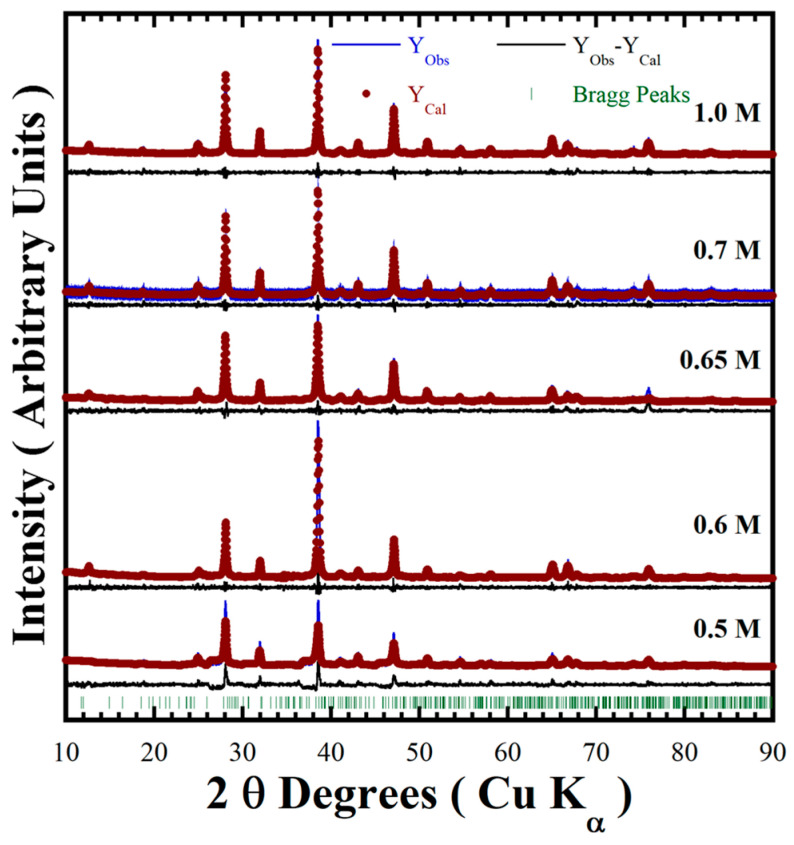
Rietveld refinement plot of BaCr_2_(P_2_O_7_)_2_ powders prepared with 50 mmol/dm^3^ of urea at 170 °C for 6 h, varying the NaOH concentration. The Bragg lines correspond to the triclinic structured pyrophosphate (MP card no. 1192170) [19].

An additional feature revealed from the XRD patterns of all BaCr_2_(P_2_O_7_)_2_ powders exhibited a systematic diffraction peak displacement regardless of the peak positions on the single-phase pattern corresponding to the triclinic-structured pyrophosphate (MP Card No. 1192170). Therefore, the detailed Rietveld refinements revealed differences in the samples’ unit cell parameters and the lattice strain factor (Table 2). The goodness-of-fit refinement values (GOF, χ^2^) and the continuous residual line computed from the experimental and calculated patterns indicate that the detailed structure was solved, showing the high accuracy of the refinement algorithm (Figure 5). The average BaCr_2_(P_2_O_7_)_2_ cell lattice parameters, together with standard deviation computed by the refinements, are “*a*_0_” = 5.4465 Å ± 0.0176 Å, “*b*_0_” = 7.5562 Å ± 0.0174 Å and “*c*_0_” = 7.6975 Å ± 0.0091 Å (see Table 2). These values are nearly similar to the original lattice parameters of BaCr_2_(P_2_O_7_)_2_ with triclinic structure, namely *a*_0_ = 5.4490 Å, *b*_0_ = 7.5698 Å and *c*_0_ = 7.6927 Å. These structural variations are likely due to partially incorporating the anionic OH^−^ species, which can provoke the slight displacement observed on the XRD pattern of samples prepared via urea-assisted synthesis [20,24]. The reaction pathway of BaCr_2_(P_2_O_7_)_2_ under hydrothermal conditions is analogous to that of uvarovite silicate-based pigments (Ca_3_Cr_2_Si_3_O_12_) in highly concentrated alkaline hydrothermal media (NaOH 2.5–5.0 M) [18]. Therefore, the H_4_O_4_ anion units may be incorporated into the O_2_ positions at polyhedral P_2_O_7_^4−^ units; thereby, the systematic variation on both unit cell lattice parameters and the lattice strain is caused by the partial incorporation of OH^−^.

**Figure 6:**
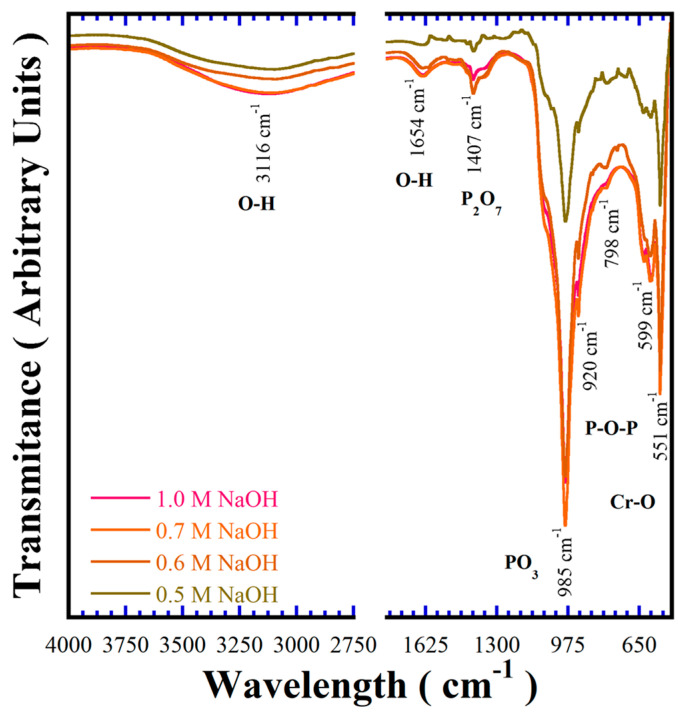
Fourier transform infrared (FT–IR) spectrum of BaCr_2_(P_2_O_7_)_2_ pigment powders crystallised at 240 °C for 24 h with 50 mmol/dm^3^ of urea using different concentrations of NaOH solution.

The BaCr_2_(P_2_O_7_)_2_ crystalline structural features were also investigated by FT–IR spectroscopy in the wavelength range of 400–4000 cm^−1^, to reveal aspects of the chemical bonding of the pigments. Figure 6 shows typical FT–IR spectra of samples prepared at 240 °C for 24 h in different concentrations of NaOH solutions in the presence of 50 mmol/dm^3^ of urea. The presence of the polyhedral P_2_O_7_^4−^ anion is associated with the vibrations of the P–O–P and P–O bonds, respectively [1]. The band due to the P–O bond in the single PO_3_^−^ tetrahedra is larger than that due to the P–O–P bridge associated with polyhedral P_2_O_7_^4−^; it is important to emphasise that this behaviour is caused by the preferential crystal growth of the particles that proceeds in the triclinic structure (112) plane, thus, a preferential aligning of the PO_3_^−^ units in these specific positions markedly promotes a large transmission peak at 985 cm^−1^. The symmetric and asymmetric stretching bands of P_2_O_7_^4−^ and P–O bonds are typically observed at 1407 cm^−1^ and 985 cm^−1^, respectively. Moreover, the symmetric and asymmetric bridge stretching modes for the P–O–P bonds constituting the polyhedral P_2_O_7_^4−^ units were revealed at 920 cm^−1^ and 798 cm^−1^, respectively [25,26]. These analyses demonstrate the existence of the paired PO_3_^−^ tetrahedral in the triclinic BaCr_2_(P_2_O_7_)_2_ powders prepared under hydrothermal conditions assisted by urea addition.

Additionally, bands within the 400 and 650 cm^−1^ range correspond to Cr–O bond vibrations [27]. The signal detected in the range between 2650–3700 cm^−1^ is attributed to water molecules adsorbed on the surface of the BaCr_2_(P_2_O_7_)_2,_ which is commonly promoted by the buffer solution being saturated in OH^−^ ions. The gradual increase in the alkalinity of the solution caused a slight increase in the content of the OH^−^ molecules in the structure of pyrophosphate pigment, which is ascribed to the lattice parameter variations [18]. The polyhedral P_2_O_7_^4−^ units occur as the principal constituent of the pyrophosphate crystalline structure, along with the OH^−^ molecules structurally adsorbed. The content of OH^−^ in the BaCr_2_(P_2_O_7_) powders was determined by TG-DSC thermal analysis (Section SF5 in SSIF), and the maximum OH^−^ amount in the oxygen positions at the polyhedral P_2_O_7_^4−^ units was 1.4 molar%. Thus, the compound’s chemical formula is BaCr_2_(P_2_O_6.9_OH_0.1_)_2_.

### 3.4. Microstructural Analysis of BaCr_2_(P_2_O_7_)_2_ Pigments Produced Under Hydrothermal Conditions

**Figure 7:**
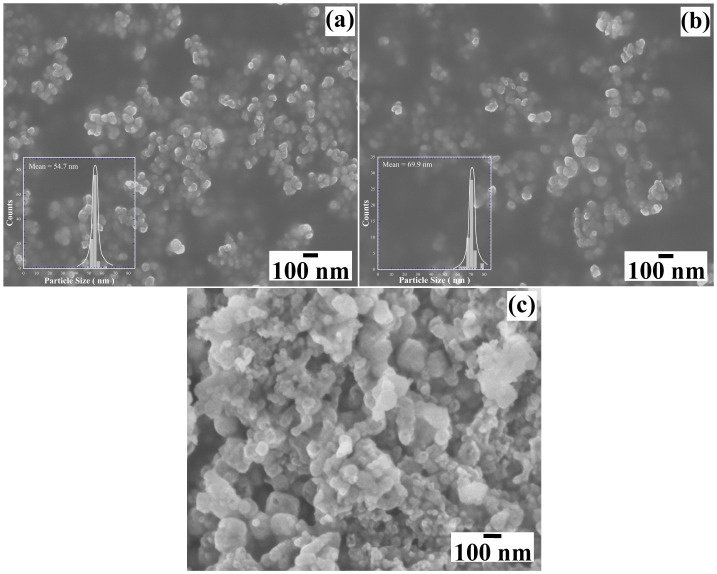
FE–SEM micrographs of the BaCr_2_(P_2_O_7_)_2_ particles produced with 50 mmol/dm^3^ of urea at different NaOH concentrations of (**a**) 0.5 M, (**b**) 0.7 M, and (**c**) 1.0 M. The treatments were carried out at 240 °C for 24 h.

Figure 7 shows the FE–SEM micrographs of the reaction products obtained in different concentrations of NaOH solutions (0.5 M, 0.7 M and 1.0 M) in the presence of 50 mmol/dm^3^ of urea at 240 °C for 24 h. Generally, the primary mesocrystals of the powders prepared in 0.5 M NaOH solution have a pseudo-spherical morphology (Figure 7a) and an average particle size of 54.7 nm ± 1.2 nm. The BaCr_2_(P_2_O_7_)_2_ particles prepared in <0.7 M NaOH solution did not change remarkably in their morphology regardless of NaOH concentrations (Figure 7b). The particles are seemingly monodispersed, showing a sharp monomodal distribution with an average size of 69.9 nm ± 1.4 nm. The increase in the OH^−^ ions concentration in the hydrothermal medium provoked a slight particle growth of the BaCr_2_(P_2_O_7_)_2_ primary mesocrystals. Interestingly, a particle agglomeration occurred in the >1.0 M NaOH solution, resulting in irregular-shaped agglomerates with a size of approximately 110 nm. This sample exhibited a bimodal size distribution: a reduced number of small particles with pseudocubic-shaped morphology had an average particle size of 75.0 ± 9.4 nm (Figure 7c). The differences in the BaCr_2_(P_2_O_7_)_2_ pigment particles’ morphology are likely provoked by the precursor gel’s rapid dissolution, leading to the agglomeration of primary mesocrystals due to accelerated particle growth. The small particles may crystallise from the remaining solute, supersaturating the NaOH solution without particle coarsening due to the fast decrease in the hydrothermal medium’s temperature during the last step of the treatment.

**Figure 8:**
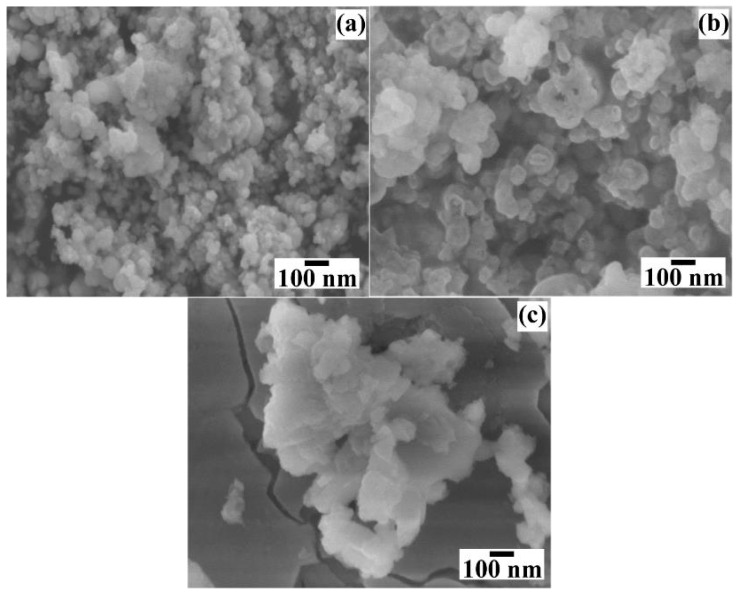
FE–SEM micrographs of BaCr_2_(P_2_O_7_)_2_ powders produced with 50 mmol/dm^3^ of urea and 0.7 M of NaOH solution at 240 °C for different reaction times of (**a**) 6, (**b**) 24, and (**c**) 48 h.

Figure 8 shows the micrographs of the BaCr_2_(P_2_O_7_)_2_ powders prepared for different reaction times. The particles produced in short reaction times (<24 h) did not show marked differences in their quasi-spherical-like morphology (Figure 8a,b), while the ones produced in longer reaction times (>48 h) had a different morphology as thin platelet-shaped particles with an average length size of 309.26 nm ± 12.9 nm (Figure 8c). Such platelet-shaped particles could be recrystallised from the solute-saturated solution generated from the dissolution of the quasi-spherical mesocrystals produced during early and intermediate reaction steps (before 24 h). This particular behaviour is caused by the low chemical stability of the BaCr_2_(P_2_O_7_)_2_ particles and the variation in the chemical composition of the solution due to the partial dilution caused by the precursor gel’s dehydration reaction, which occurred before the gel dissolution–crystallisation process. Furthermore, the morphological differences in the particle size and distribution can change in their colour CIELab coordinates and NIR reflectance behaviour [18], as discussed in Section 3.5.

**Figure 9:**
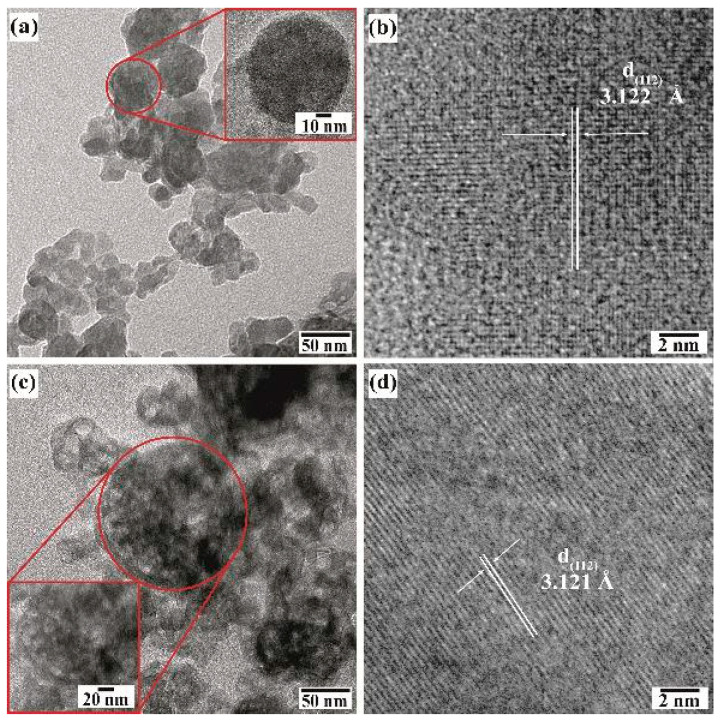
TEM (**a**,**c**) and HR-TEM (**b**,**d**) micrographs of BaCr_2_(P_2_O_7_)_2_ particles prepared with urea at 240 °C for 24 h at different NaOH concentrations of (**a**,**b**) 0.5 M and (**c**,**d**) 1.0 M. The inset micrograph corresponds to the area in the red circle.

Further details of the BaCr_2_(P_2_O_7_)_2_ particle morphology were investigated by HR-TEM (Figure 9). Even though the pigment powders had a homogeneous quasi-spherical morphology according to FE-SEM, irrespective of the concentration of NaOH in the preparation (Figure 7a,b), the particles obtained in the 0.5 M NaOH solution consisted of euhedral fine crystals and quasi-spherical mesocrystals according to the TEM images (Figure 9a,b). The quasi-spherical aggregates could be produced by partially dissolving the finest euhedral particles with the solute recrystallised almost in the same place. The remaining euhedral crystals bonded with each other to form a 3D architecture with some tiny pores, as depicted in the inset micrograph of the pseudo-spherical mesocrystal in Figure 9a. Irregular nodular-shaped particle recrystallisation was triggered in the highly concentrated 1.0 M NaOH solution, producing the irregular nodular mesocrystals as seen in Figure 9c. The highly OH-concentrated solvent accelerates the euhedral pigment particles’ dissolution rate, resulting in spontaneous solute recrystallisation which leads to random formation of bulky irregular nodular-shaped mesocrystals. As can be seen from the HR-TEM images (Figure 9b,d), the recrystallised mesocrystals exhibited a high crystallinity, as depicted by the well-defined atomic stacking ordering in both samples prepared in the NaOH solution with different concentrations. Interestingly, the lattice fringe interplanar spacing is similar in both samples, averaging 3.123 Å, which was indexed with the plane with Miller indexes (112) corresponding to the triclinic structure of BaCr_2_(P_2_O_7_)_2_ [19]. The images indicate that the mesocrystals’ recrystallisation occurred preferentially along the {112} crystallographic family plane, which corresponds to the peak at a 2θ angle of 38.22° in the XRD patterns in Figure 3. Therefore, the pseudo-spherical pyrophosphate mesocrystals should be formed by a bulky 3D hierarchical self-assembly that proceeds along a specific direction, which is analogous to the process for producing other inorganic cold pigments prepared under hydrothermal conditions [16,18].

### 3.5. BaCr_2_(P_2_O_7_)_2_ Pigments Colour and NIR Solar Reflectance Analyses

The colour of the hydrothermally prepared BaCr_2_(P_2_O_7_)_2_ pigments was determined by CIELab* measurements using the UV–Vis spectroscopy colourimetry technique. The chroma value was calculated using the mathematical expression ${C_{ab}}^{*}=\sqrt{{\left(a^{*}\right)}^{2}+{\left(b^{*}\right)}^{2}}$ [28]. Table 3 summarises the chromatic coordinates L*a*b* and the chroma values determined for various pigments prepared under different experimental conditions. The RGB colour coordinates, obtained by converting the L*a*b* values and the pigment colour tonality associated with their RGB coordinates, are also given in Table 3. In general, the colour of the powder pigment falls within the standard CIELab coordinates of the pyrophosphate BaCr_2_(P_2_O_7_)_2_ green pigment. However, the hue is brighter than the sample prepared by solid-state reaction at high temperature (L = 72.5, a* = −20.5, b* = 14.7 and C_ab_* = 25.22) [1].

On the other hand, the single-phase pigment’s b* coordinate exhibited negative values, which caused a bright green hue in most of the samples prepared with urea under alkaline hydrothermal conditions. The pigment’s chroma (C_ab_*) is consistent with the CIELab coordinates, with only a variation within the range of 8.89 to 15.50. According to these results, the change in green colour tonality determined on the powders prepared under different treatment conditions strongly depends on the inherent microstructural parameters, such as particle size and morphology. Indeed, the increase in the OH^−^ ions in the alkaline solution provoked the growth of BaCr_2_(P_2_O_7_)_2,_ the pseudo-spherical-shaped particles during the first step. Subsequently, mesocrystals produced by recrystallisation during the hydrothermal processing led to a slight particle size increase of approximately 25.5 nm. Even the slight dimensional change from the tiny increase in the particle size physically increased the brightness of BaCr_2_(P_2_O_7_)_2_ powders, resulting in low chroma values related to the pigment’s dull and muted colour tonality. This behaviour is analogous to the phenomenon inherent in powder pulverisation, where the particle size reduction enhances an optimum interaction between the surface area and the sunlight, resulting in a light and bright colour tonality. Furthermore, the particle morphology is less dependent on the pigments’ colour intensity and colour saturation, even if the particle size of pseudo-spherical (C_ab_* = 9.80) intrinsic morphologies is considerably different from that of plate-like (C_ab_* = 15.80) ones, resulting in vibrant green tonalities in the recrystallised BaCr_2_(P_2_O_7_)_2_ plate-like particles.

**Figure 10:**
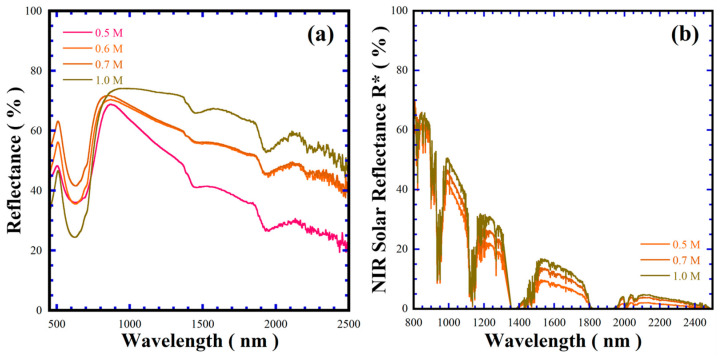
(**a**) Visible-NIR spectral reflectance and (**b**) solar reflectance spectra of BaCr_2_(P_2_O_7_)_2_ powders prepared with the assistance of urea addition at 240 °C for 48 h, varying the NaOH solution concentration.

Near infrared (NIR) radiation comprises 52% of the total solar light spectrum, which gives rise to the urban heat island phenomenon. Thus, several research groups have developed cold pigments to decrease electric consumption during air conditioning and suppress the heat generated by the absorption of NIR radiation in solids [29,30]. The optical properties in the NIR, regarding the band gap and solar reflectance (% R*), were investigated. The Kubelka–Munk function provided the BaCr_2_(P_2_O_7_)_2_ pigment bandgap values given in Table 3; the average pigment band gap is 3.12 ± 0.03 eV (the plots are shown in Figure S7 in SSIF), while the solar reflectance showed a marked difference between 55.41 and 67.46%. The maximum solar reflectance of the BaCr_2_(P_2_O_7_)_2_ pigment is 10.0% lower than that of the Rutile TiO_2_ standard (79.01%, <5 mm in size), considered as an adequate cold pigment. According to the microstructural differences in Table 3 and Figure 8 and Figure S5 in SSIF, the optical properties depend on the particle size and morphology, rather than the free carrier density mechanism that is argued to increase the reflectivity of an inorganic pigment [31,32,33]. Indeed, the larger-sized BaCr_2_(P_2_O_7_)_2_ powders provided larger specular reflectance behaviour from 300 nm to 2500 nm, as shown in Figure 10.

On the other hand, the broad absorption peak observed at a wavelength of approximately 522 nm for all the samples falls within the reflectance range of other inorganic green pigments reported elsewhere [34]. As shown in Figure 10a, the peak increased slightly in intensity with increasing alkaline concentration in the hydrothermal medium. This variation might be associated with differences in particle size, which influence the optical properties of the pigment, particularly the a* and b* chromaticity coordinates, resulting in the variation of the pigment’s colour hue property. In addition, the differences in the BaCr_2_(P_2_O_7_)_2_ pigment colour CIELab* coordinates with those of other green pigments containing the Cr^3+^ chromophore are discussed in Section SF6 in the SSIF.

On the other hand, Figure 10 shows the NIR reflectance spectrum and the NIR solar reflectance for the BaCr_2_(P_2_O_7_)_2_ pigments with different particle size and morphology produced in various concentrations of NaOH solution in the presence of 50 mmol/dm^3^ urea at 240 °C for 48 h, in the range of 300–2500 nm calculated according to ASTM G173-03 [35]. The control of particle size and morphology of the BaCr_2_(P_2_O_7_)_2_ pigments could optimise their reflectance, based on the results in Figure 10 and Table 3. In general, the pigment with pseudo-spherical-shaped morphology exhibited the lowest spectral reflectance within 800–2500 nm as well as the lowest reflectance within 800–1500 nm, averaging a NIR solar reflectance of 60.0% as revealed at 800 nm (Figure 10a,b). The change in the particle morphology to agglomerated mesocrystals results in a decrease in the spectral reflectance to a minimum of 40% at 2500 nm; these results indicate that the reflectance decrease is irrespective of the particle size rather than the particle morphology. Indeed, the variation in the pigment’s reflectance is analogous to that on the samples with a small particle size (44.3–75.07 nm), Figure S8 in the SSFI. Interestingly, the decrease in reflectance of mesocrystal powder is similar to that of the TiO_2_ powder in the wavelength range of 800–1700 nm; the reflectance difference is approximately 7.5%. The plate-like shaped BaCr_2_(P_2_O_7_)_2_ powder improved in reflectance by nearly 20% in the NIR spectra (800–2500 nm), as shown in Figure 10a, and the maximum spectral solar reflectance (67.46%). Solar spectral reflectance can be related to the particle surface aspect: flatter and more faceted particle surfaces provoke an optimum reflection of NIR spectrum photons. Moreover, the NIR solar reflectance capability of the hydrothermally prepared BaCr_2_(P_2_O_7_)_2_ pigment is 10% lower than that of the one prepared via solid-state reaction [1]. Since the reported functional cold pigment inorganic oxides exhibited bulk solar reflectance with R* values of above 35.0%, such as the spinel-structured ZnCr_2_O_4_, CoCr_2_O_4_ and CoCrAlO_4_ prepared by sol-gel calcination [28,29,30,31,32,33,34]. The solar reflectance values of these pigments are summarised in Table S2 in the SSIF. The differences in the green pigments’ solar reflectance are discussed in Section SF6 of the SSIF. According to this evaluation, we surmise that the BaCr_2_(P_2_O_7_)_2_ pigment hydrothermally prepared here has great potential for cold pigment applications based on the spectral reflectance and the NIR solar reflectance behaviour in Figure 10a,b, and the NIR solar reflectance in Table S2 in SSIF.

## 4. Conclusions

The pyrophosphate pigment BaCr_2_(P_2_O_7_)_2_ has been successfully prepared under alkaline hydrothermal conditions in the presence of urea, used as a reducing agent. The formation of single–phase BaCr_2_(P_2_O_7_)_2_ with triclinic structure was triggered via a single-step reaction of the precursor gel achieved by dissolution–crystallisation at a pH range of 9.46–10.53. The polyatomic HCO_3_^−^ ions from urea decomposition provided a buffered, mildly alkaline reaction environment, leading to the crystallisation of single-phase BaCr_2_(P_2_O_7_)_2_ pigment without byproducts at a low temperature, 170 °C, for a short reaction time of 6 h. Fine pyrophosphate particles with a size averaging 55.0 ± 10.0 nm and a quasi-spherical morphology were preferentially crystallised by the initial single-step reaction at temperatures below 240 °C for 24 h. The BaCr_2_(P_2_O_7_)_2_ powders underwent a secondary dissolution–recrystallisation process favoured by the NaOH solution concentration increase (0.5–1.0 M). Under these conditions, a marked morphology variation occurred in the BaCr_2_(P_2_O_7_)_2_ recrystallised particles, namely quasi-spherical (133.8 nm), nodular-like mesocrystals (221.7–309.3 nm) and plate-like shapes (342.5 nm). The differences in the morphology of BaCr_2_(P_2_O_7_)_2_ pigments improved the NRI spectral reflectance (57.7–67.5%) and NIR solar reflectance (55.4–67.5%). Plate-like shaped particles exhibited the optimum NIR reflectance, due to their flat faceted surfaces rather than their particle size. Therefore, the solar reflectance capability of BaCr_2_(P_2_O_7_)_2_ provides great potential for eco–friendly applications such as the preparation of thermally reflective coatings, synergistically contributing to the sustainable development of pigments to mitigate the urban heat island phenomenon.

## Figures and Tables

**Table 1:** Atomic coordinates and Wyckoff positions of the BaCr_2_(P_2_O_7_)_2_ with triclinic structure (MP card no. 1192170, space group *P*–*1*, 2) were used to carry out the Rietveld refinements by TOPAS 4.2 software.

Element	Wyckoff Position	Occupation	Spatial Coordinates (BaCr_2_(P_2_O_7_)_2_)		
			*x*/*a*	*y*/*b*	*z*/*c*
Ba	1a	1	0.78918	0.72322	0.06838
Cr	2i	1	0.80807	0.85331	0.59828
P1	2i	1	0.28179	0.68455	0.39885
P2	2i	1	0.29919	0.76506	0.78656
O1	2i	1	0.54268	0.77864	0.39328
O2	2i	1	0.26785	0.62503	0.59072
O3	2i	1	0.28371	0.65099	0.92607
O4	2i	1	0.22382	0.51389	0.25542
O5	2i	1	0.91599	0.10993	0.19970
O6	2i	1	0.44831	0.12507	0.20392
O7	2i	1	0.91709	0.17587	0.59447

**Table 2:** Summary of the experiments proposed to investigate the preparation of BaCr_2_(P_2_O_7_)_2_ powders under hydrothermal conditions employing various solutions at different temperatures and reaction times.

Sample ID	Temperature (°C)	Time (h)	V H_2_O (mL)	NaOH * (M)	Urea (mmol/dm^3^)	Initial pH	Final pH	Phases Determined by XRD (wt.%)			Lattice Parameters			Lattice Strain	GOF
								Amorphous Phase	BaCr_2_(P_2_O_7_)_2_	BaHCr_2_PO_10_	a_0_ (Å)	b_0_ (Å)	c_0_ (Å)		
BCPN01	170	48	12.5	-	-	6.17	6.05	100	-	-	-	-	-		-
BCPN02	200		12.5	-	-	6.14	5.94	100	-	-	-	-	-		-
BCPN03	240		12.5	-	-	6.15	5.9	100	-	-	-	-	-		-
BCPN05	240	48	-	0.5	-	12.57	12.66	-	61.8 (5)	38.1 (5)	5.4563 (10)	7.5494 (10)	7.6797 (10)	0.78 (0.2)	0.9
BCPN06			-	0.6	-	12.8	12.88	-	62.2 (4)	37.7 (4)	5.4380 (10)	7.5749 (10)	7.7038 (10)	1.21 (0.1)	0.9
BCPN07			-	0.65	-	12.89	12.99	-	69.9 (1)	30.0 (1)	5.4564 (10)	7.5550 (20)	7.6932 (20)	1.56 (0.1)	0.9
BCPN08			-	0.7	-	12.98	13.08	-	76.9 (1)	24.0 (0)	5.4334 (10)	7.5772 (30)	7.7070 (20)	1.88 (0.1)	0.9
BCPN09			-	1.0	-	13.59	13.61	-	74.0 (1)	26.9 (1)	5.4503 (20)	7.5497 (20)	7.6976 (30)	1.95 (0.1)	0.8
BCPN41			-	0.7	25	12.96	10.66	-	69.2 (2)	30.7 (1)	5.4632 (30)	7.5745 (10)	7.6869 (10)	1.80 (0.1)	0.8
BCPN43			-	0.7	50	12.96	10.44	-	100	-	5.4697 (30)	7.5557 (20)	7.6988 (20)	1.81 (0.1)	1.1
BCPN44			-	0.7	100	12.89	9.96	88.6 (1)	11.3 (1)	-	5.4690 (10)	7.5782 (10)	7.6988 (10)	1.87 (0.1)	1.0
BCPN45			-	0.7	150	12.88	9.7	100	-	-	-	-	-	-	-
BCPN46			-	0.7	200	12.87	9.46	100	-	-	-	-	-	-	-
BCPN47			-	0.7	75	12.95	10.01	100	-	-	-	-	-	-	-
BCPN90	240	6	-	0.5	50	12.49	10.16	-	100	0	5.4615 (10)	7.5440 (10)	7.6828 (10)	1.21 (0.1)	1.1
BCPN93			-	0.7	50	12.87	10.53	--	100	0	5.4335 (20)	7.5472 (10)	7.6811 (20)	1.72 (0.2)	1.1
BCPN105		24	-	0.5	50	12.53	10.23	-	100	0	5.4250 (10)	7.5815 (20)	7.7036 (20)	1.17 (0.1)	1.1
BCPN108			-	0.7	50	12.87	10.49	-	100	0	5.4432 (10)	7.5397 (20)	7.7092 (30)	1.99 (0.2)	1.1
BCPN110	200	6	-	0.5	50	12.52	10.21	-	100	0	5.4472 (10)	7.5306 (30)	7.7022 (20)	1.23 (0.1)	0.8
BCPN107			-	0.7	50	12.75	10.49	-	100	0	5.4235 (30)	7.5472 (30)	7.7026 (40)	1.82 (0.1)	0.8
BCPN115	170		-	0.5	50	12.49	10.34	-	100	0	5.4629 (10)	7.5720 (20)	7.6949 (20)	1.25 (0.1)	0.9
BCPN100			-	0.7	50	12.71	10.52	-	100	0	5.4496 (20)	7.5747 (30)	7.7070 (30)	1.93 (0.1)	0.9

Note: * The volume of the alkaline NaOH solution used for each treatment was 12.5 mL. The lattice parameters correspond to the phase BaCr_2_(P_2_O_7_)_2_.

**Table 3:** BaCr_2_(P_2_O_7_)_2_ pigments particle size, CIELab* values, chroma, RGB coordinates, and colour hue; prepared with 50 mmol/dm^3^ of urea at different temperatures, reaction intervals and NaOH media concentrations.

Sample ID	Temperature (°C)	Time (h)	NaOH (M)	Crystalline Size (nm)	Crystalline Size * (nm)	Bandgap (eV)	Solar Reflectance (% R*)	CIELab Coordinates			RGB Colour Coordinates			Chroma C_ab_*	Colour Hue
								L*	a*	b*	R	G	B		
BCPN90	240	6	0.5	47.3 (6.2)	42.0 (1.1)	3.16	57.94	66.12	−7.94	−4.58	137	166	170	9.16	
BCPN91			0.6	53.8 (1.2)	54.1 (6.1)	3.11	58.99	67.58	−9.84	−1.94	138	171	168	10.03	
BCPN92			0.7	57.1 (1.8)	57.8 (2.8)	3.09	59.76	68.72	−10.49	−2.31	139	175	172	10.75	
BCPN93			1.0	65.3 (1.1)	62.3 (6.2)	3.09	60.40	70.99	−10.32	−3.94	144	181	182	11.05	
BCPN105		24	0.5	54.7 (1.1)	55.7 (1.1)	3.18	62.93	65.48	−9.00	−4.08	133	165	168	9.88	
BCPN106			0.6	66.0 (1.2)	60.2 (2.1)	3.13	63.60	68.22	−9.88	−3.14	138	173	173	10.36	
BCPN107			0.7	69.9 (1.4)	66.3 (1.2)	3.10	65.23	68.07	−13.21	−3.11	129	175	172	13.57	
BCPN108			1.0	75.0 (9.3)	72.5 (1.4)	3.08	67.03	72.20	−15.25	−2.85	134	187	183	15.51	
BCPN120		48	0.5	133.8 (50.1)	51.3 (1.1)	3.16	61.72	73.87	−9.24	−3.95	154	188	190	10.05	
BCPN121			0.6	221.7 (40.5)	60.6 (9.3)	3.13	63.76	69.78	−12.94	−1.86	143	178	174	13.07	
BCPN122			0.7	309.2 (102.8)	62.4 (6.1)	3.09	66.05	67.29	−14.59	−2.54	133	174	171	14.80	
BCPN123			1.0	342.4 (151.7)	64.1 (6.1)	3.09	67.46	66.41	−15.48	−2.74	129	172	169	15.72	
BCPN110	200	24	0.5	54.6 (4.5)	53.2 (8.9)	3.15	61.28	67.01	−8.86	−3.32	141	167	169	9.46	
BCPN111			0.6	60.7 (2.0)	59.8 (5.1)	3.11	63.53	68.22	−12.08	−2.42	137	170	168	12.32	
BCPN112			0.7	63.5 (6.2)	60.7 (1.1)	3.11	64.28	58.96	−12.88	−3.14	137	174	173	13.25	
BCPN113			1.0	71.8 (1.9)	64.7 (1.3)	3.09	66.74	65.29	−15.30	−2.50	108	150	147	15.50	
BCPN114	170		0.5	50.6 (4.6)	54.8 (0.9)	3.18	55.41	65.29	−7.55	−4.70	139	163	169	8.89	
BCPN115			0.6	60.6 (5.2)	62.5 (0.9)	3.13	57.73	65.2	−7.54	−4.75	139	163	169	8.91	
BCPN116			0.7	62.1 (1.5)	67.2 (0.9)	3.11	58.88	65.66	−8.06	−4.82	139	164	170	9.39	
BCPN117			1.0	70.5 (1.4)	79.5 (3.4)	3.08	59.06	63.5	−9.12	−3.60	133	159	161	9.80	
TiO_2_	-	-	-	<5000	-		79.01	97.85	0.32	1.65	251	249	246	1.68	

Note: * Crystalline size values were calculated using the Rietveld refinement method. The colour hue corresponds to the RGB coordinates measured for each sample.

## Data Availability

Data will be made available upon request.

## Supplementary Materials

The following supporting information can be downloaded at: https://www.mdpi.com/article/10.3390/nano15130982/s1, Figure S1: X-ray diffraction patterns of BaCr_2_(P_2_O_7_)_2_ powders prepared under hydrothermal conditions with a concentration of 50 mmol/dm^3^ of urea and 0.7 M NaOH at 240 °C for different reaction times; Figure S2: DSC and TGA curves of the precursor gel and pyrophosphate powders (BaCr_2_(P_2_O_7_)_2_) prepared under hydrothermal conditions at 240 °C for 24 h with 50 mmol/dm^3^ of urea, varying the NaOH solution concentration; Figure S3: XPS survey spectrum of the hydrothermally prepared green pigment powders (a) without urea and (b) with urea; Figure S4: Core-level Cr 2p and O 1s high-resolution XPS spectrum of the hydrothermally prepared green pigment powders; Figure S5: FE-SEM micrographs of the BaCr_2_(P_2_O_7_)_2_ pigments produced under hydrothermal conditions with 50 mmol/dm^3^ of urea at 240 °C for 48 h in NaOH solutions with concentrations of a) 0.5 M, b) 0.6 M, and c) 1.0 M; Figure S6. Differential scanning calorimetry and thermogravimetry curves of the precursor gel and pyrophosphate powders (BaCr_2_(P_2_O_7_)_2_) prepared at 240 °C for 24 h with 50 mmol/dm^3^ urea, varying the NaOH solution concentration; Figure S7: Kubelka–Munk curves of BaCr_2_(P_2_O_7_)_2_ pigments prepared under hydrothermal conditions with 50 mmol/dm^3^ at 240 °C for 48 h, varying concentration of NaOH solution; Figure S8: UV-vis NIR spectral reflectance curves and b) NIR solar reflectance spectra of BaCr_2_(P_2_O_7_)_2_ pyrophosphate pigments prepared under hydrothermal conditions at 240 °C for 24 h by varying the NaOH concentration and Rutile (TiO_2_), Table S1: CIF file of the secondary crystalline phase determined in the XRD patterns of the powders prepared in hydrothermal conditions.
