# Supplementary material for: Fast Alkaline Hydrothermal Synthesis of Pyrophosphate BaCr2(P2O7)2 Nanoparticles and Their NIR Spectral Reflectance
Source: Nanomaterials (Basel). 2025 Jun 25;15(13):982. doi: 10.3390/nano15130982 (PMC12251539; doi:10.3390/nano15130982)
Supplement: Supplementary file 1 [file nanomaterials-15-00982-s001.zip › nanomaterials-3659092-supplementary.pdf]

# Supplementary Supporting Information

## Fast Alkaline Hydrothermal Synthesis of Pyrophosphate $BaCr_2(P_2O_7)_2$ Nanoparticles and Their Reflectance in the NIR

**D.E. Carrillo–Ramírez[1], J.C. Rendón–Angeles[1,*], Z. Matamoros–Veloza[2], J. López – Cuevas[1], I. Juárez – Ramírez[3] and T. Ueda[4]**

[1]*Centre for Research and Advanced Studies of the National Polytechnic Institute, Saltillo Campus, Ramos Arizpe, 25900, Coahuila, México.*

[2]*Tecnológico Nacional de México /(I.T. Saltillo), Technological Institute of Saltillo, Graduate Division, Saltillo 25280, México.*

[3]*Faculty of Civil Engineering–Department of Ecomaterials and Energy; Cd. Universitaria, San Nicolás de Los Garza, Nuevo León 66455, México.*

[4]*Department of Marine Resources Science, Faculty of Agriculture and Marine Science and Centre for Advanced Marine Core Research, Kochi University, Nankoku 783–8502, Japan.*

***SF1 Crystalline structural and optical characterisation***

The cards of the crystalline phases determined in the reaction products' XRD patterns are shown in Tables S1-S2, and these cards were taken from the COD 2014 database and the High Score Plus Panalytical software version 3.0. The crystallographic data were included in subroutines of the algorithm designed to carry out the structural refinement. A Chebyshev 10-coefficient polynomial function modelled the background shifting, while a pseudo–Voigt function refined the peak shape. The refinement algorithm approach calculated the crystalline structural parameters: unit lattice cell, isotropic thermal displacement, crystallite size, and the residual amount of the pyrophosphate powder and the secondary phase.

**Table S1** Atomic coordinates of the $BaHCr_2PO_{10}$ with the triclinic structure used to carry out the Rietveld refinement by TOPAS 4.2 software; the spatial locations were reported previously elsewhere in the CIF file, COD card no. 96-901-6408.

| Element identification | Wyckoff position | Occupation | Spatial coordinates ($BaHCr_2PO_{10}$) | | |
|---|---|---|---|---|---|
| | | | *x/a* | *y/b* | *z/c* |
| Ba | 2i | 1 | 0.16567 | 0.26464 | 0.83272 |
| Cr1 | 2i | 1 | 0.30270 | 0.09770 | 0.40810 |
| Cr2 | | | 0.38400 | 0.78510 | 0.00390 |
| P1 | 2i | 1 | 0.20210 | 0.35730 | 0.20020 |
| O1 | 2i | 1 | 0.10340 | 0.37640 | 0.09300 |
| O2 | 2i | 1 | 0.10730 | 0.52820 | 0.29790 |
| O3 | 2i | 1 | 0.17450 | 0.20670 | 0.28780 |
| O4 | 2i | 1 | 0.41800 | 0.32960 | 0.14870 |
| O5 | 2i | 1 | 0.53500 | 0.01700 | 0.32430 |
| O6 | 2i | 1 | 0.78700 | 0.05000 | 0.53120 |
| O7 | 2i | 1 | 0.27100 | 0.23610 | 0.52930 |
| O8 | 2i | 1 | 0.46100 | 0.74900 | 0.13520 |
| O9 | 2i | 1 | 0.21700 | 0.70670 | 0.01420 |
| O10 | 2i | 1 | 0.68900 | 0.01090 | 0.01330 |
| O11 | 2i | 1 | 0.69500 | 0.46480 | 0.28550 |
| O12 | 2i | 1 | 0.93600 | 0.12000 | 0.23780 |
| O13 | 2i | 1 | 0.29400 | 0.60150 | 0.44140 |

The $BaCr_2(P_2O_7)_2$ pigments' bandgap energy values corresponded to the ultraviolet spectral reflectance (300–700 nm) data. The Kubelka-Munk function (Eq. S1) was used to obtain the plot shown in Fig. S3. Figure S3 portrays the bandgap plot of the reaction products prepared at 240 °C for 48 h with a concentration of 50 mmol/dm$^3$ of urea varying the NaOH solution concentration. These results agree with the bandgap of 3.13 eV reported in the MP card No. 1192170, confirming the semiconductor nature of the pigments. The average bandgap value of 3.12 also has a minor statistical standard deviation of ± 0.03 eV.

Kubelka–Munk equation, i.e.,

$$F(R) = \frac{1 - R^2}{2R} \qquad \text{Eq. S1}$$

where R is the observed reflectance of the samples.

The NIR Solar reflectance ($R^*$) measurements were carried out according to the ASTM standard number G173–03. The solar reflectance of the developed pigments was calculated over the wavelength range of 700–2500 nm using the Eq. S2:

$$R^* = \frac{\int_{700}^{2500} r(\lambda)\, i(\lambda) d(\lambda)}{\int_{700}^{2500} i(\lambda) d(\lambda)} \qquad \text{Eq. S2}$$

where *r(λ)* is the spectral reflectance experimentally obtained, and *i(λ)* is the solar spectral irradiance ($Wm^{-2}mm^{-1}$) obtained from ASTM standard number G173–03.

### *SF2. Characterisation of the crystalline structure of $BaCr_2(P_2O_7)_2$ pigment*

Figure S1 shows the XRD patterns of $BaCr_2(P_2O_7)_2$ powders prepared at 240 °C with a urea concentration of 50 mmol/$dm^3$ and 0.7 M NaOH varying the reaction interval. The powders prepared for 6 and 24 h exhibited a preferential growth parallel to crystallographic plane with Miller indexes of (112) at 2θ = 38.5°, this particle coarsening trend changed above 24 h. The XRD patterns of the powder prepared for 48 h exhibit a marked intensity increase for the peak located at 2θ = 28.01°, depicting the particle growth occurring parallel to the Miller index plane $(1\bar{1}\bar{2})$, which belongs to the {112} plane family. Furthermore, the peaks were slightly shifted to smaller 2θ angles. This behaviour is attributed to water molecules' incorporation in the $BaCr_2(P_2O_7)_2$ pigment triclinic structure. According to these results, we assumed that a dissolution-recrystallisation reaction might achieve the variation in the particle coarsening, which caused the enlargement of the diffraction peaks mentioned above.

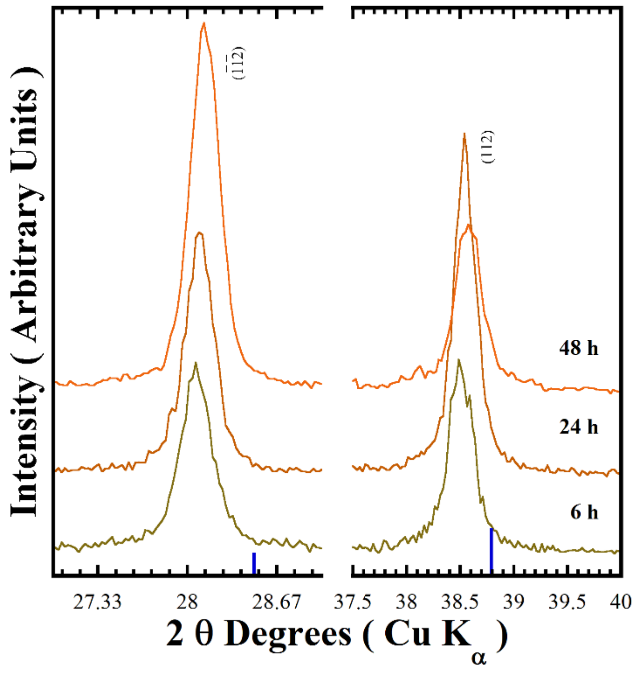


**Figure S1**. X-ray diffraction patterns of $BaCr_2(P_2O_7)_2$ powders prepared under hydrothermal conditions with a concentration of 50 mmol/$dm^3$ of urea and 0.7 M NaOH at 240 °C for different reaction times.

Figure S2 shows the typical Rietveld refinement plots of the samples prepared with the 0.5 and 0.7 M NaOH solutions under hydrothermal conditions at 240 °C for 48 h. These samples exhibited the simultaneous formation of the $BaCr_2(P_2O_7)_2$ powders and the $BaHCr_2PO_{10}$ secondary phase. These XRD patterns were computed using the reference files from the MP and COD 2014 databases included in the HighScore Plus (PANalytical) software; the CIF information corresponding to each phase is summarised in Tables 1 and S1, respectively. In addition, the quantitative phase analysis was performed using the Rietveld refinement method, which enabled the determination of phase content by fitting a calculated pattern based on crystallographic models based on least square equations to the experimental XRD powder diffraction pattern. Structural parameters were refined to minimise the difference between the observed and the calculated patterns, which results in low goodness-of-fit (GOF) values (Table 2), confirming the reliability of the refinement algorithm. According to these results, the sample prepared in the 0.5 M NaOH solution (Fig. S2a) was composed by 63.8 wt% of $BaCr_2(P_2O_7)_2$ and 36.1 wt% of $BaHCr_2PO_{10}$ secondary phase. In comparison, the sample synthesised with 0.7 M NaOH (Fig. S2b) exhibited a higher purity of the $BaCr_2(P_2O_7)_2$ phase, suggesting that increased alkalinity promotes an accelerated crystallisation of the target pyrophosphate, slightly decreasing the secondary phase formation.

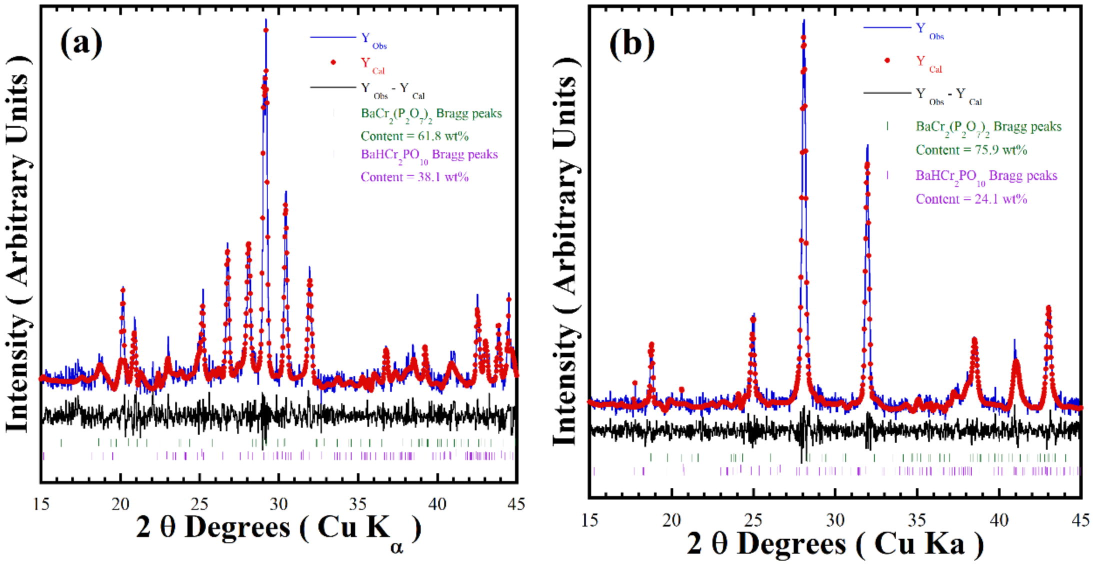


**Figure S2.** XRD patterns of powder samples prepared at 240 °C for 48 h using different NaOH solution concentrations, a) 0.5 M and b) 0.7 M. The phase contents were calculated only considering the triclinic structured crystalline phases $BaCr_2(P_2O_7)_2$ and the secondary phase $BaHCr_2PO_{10}$. These were calculated according to the crystallographic information provided by the CIF files from the COD database.

### *SF3. Chemical compositional characterisation at the surface of the $BaCr_2(P_2O_7)_2$ particles*

Figure S3 shows the typical XPS survey spectrum of the green pigment powders prepared under standard experimental conditions, 240 °C for 48 h with a 0.7 M NaOH solution (a) without the addition of urea and in the presence of 50 mmol/l of Urea. Likewise, Figure S4 show the non-deconvoluted core level Cr $2p_{3/2}$ and O 1s spectrum, which were included to demonstrate the differences between the powder samples hydrothermally produced without adding urea (dark blue dots) and in the presence of urea (claret dots). It deserves emphasising that the sample prepared without urea exhibited a large FHWM for the Cr $2p_{3/2}$ energy binding peak; this difference is caused by the crystalline phases of $BaCr_2(P_2O_7)_2$ and $BaHCr_2PO_{10}$, containing the $Cr^{3+}$ and $Cr^{6+}$ ionic species, respectively, as seen in Figure S4. In both samples, the large FHWM peaks (>2.6 eV) discard the possibility that multiple splitting peaks must apply to deconvolve the

$Cr^{3+}$ species on the spectra of the pure $BaCr_2(P_2O_7)_2$, as occurs in other chromite oxides, $FeCr_2O_4$ and $NiCr_2O_4$.

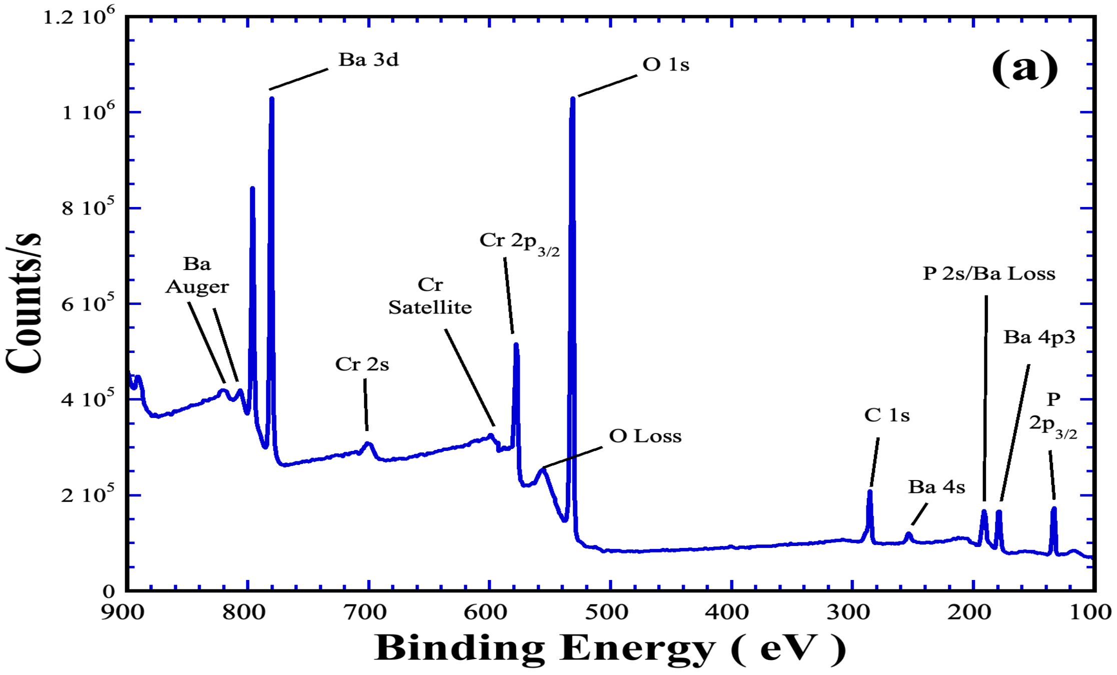


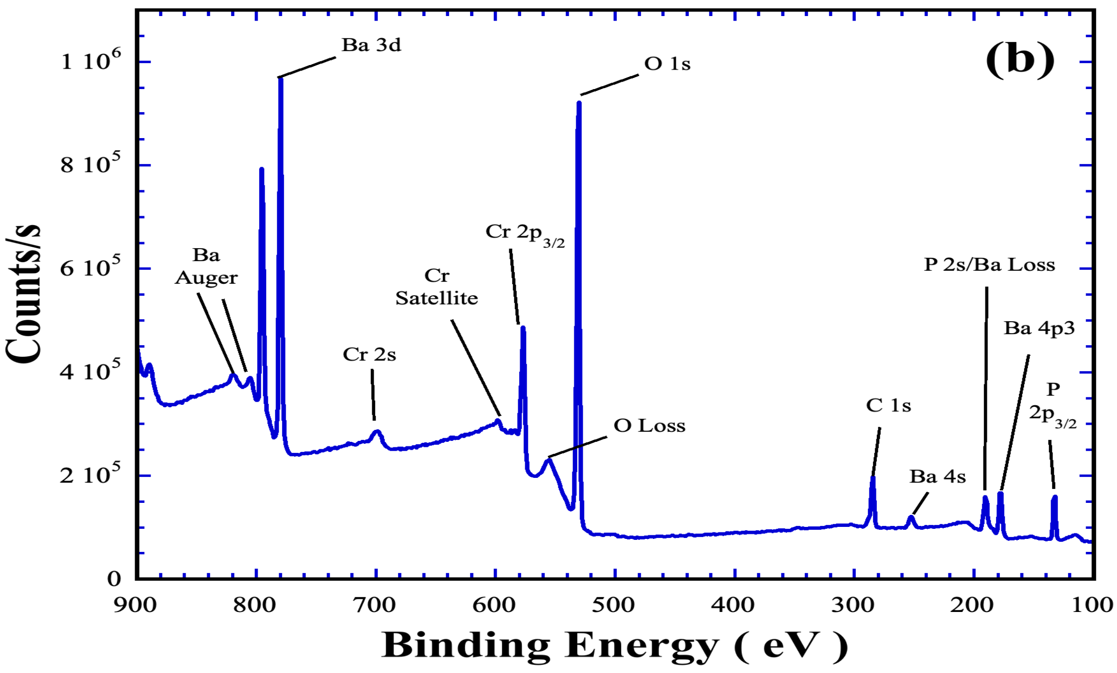


**Figure S3.** XPS survey spectrum of the hydrothermally prepared green pigment powders (a) without urea and (b) with urea.

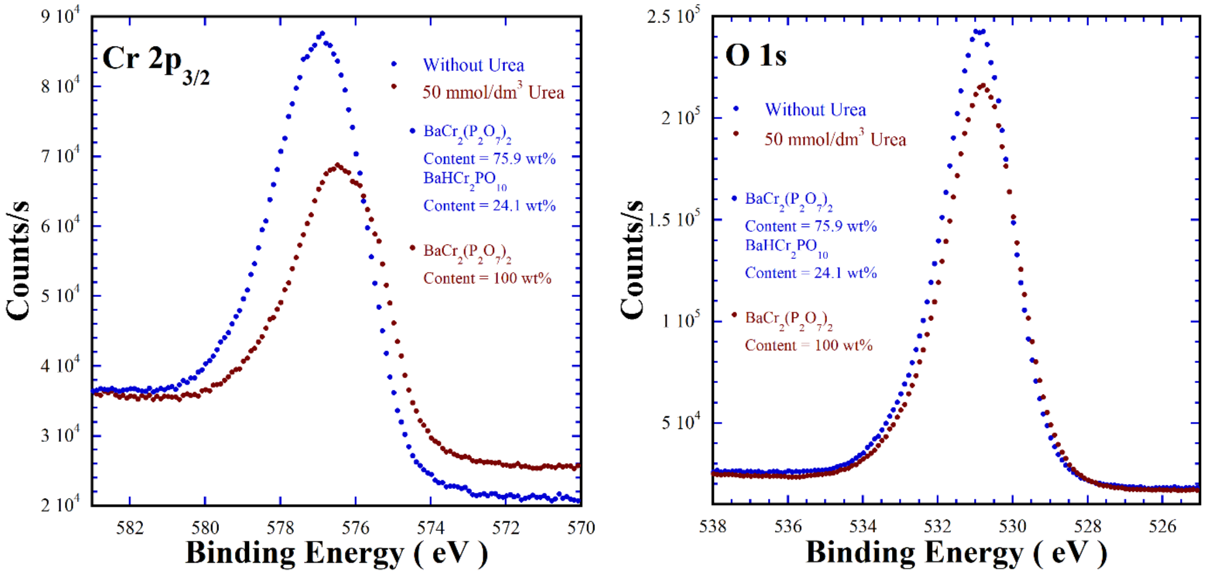


**Figure S4.** Core-level Cr $2p_{3/2}$ and O 1s high-resolution XPS spectrum of the hydrothermally green pigment powders.

***SF4 Microstructural features of the hydrothermally prepared $BaCr_2(P_2O_7)_2$ powders***

Figure S5 shows the FE-SEM micrographs of $BaCr_2(P_2O_7)_2$ pigment powders synthesised via hydrothermal treatment with 50 mmol/dm$^3$ of urea at 240 °C for 48 h varying the concentration of NaOH, a) 0.5 M, b) 0.7 M and c) 1.0 M. These observations revealed that a progressive microstructural morphology evolution is trigged by the increase of the alkalinity of the hydrothermal fluid. The particles prepared at 0.5 M NaOH exhibited a bimodal distribution constituted by quasi-spherical primary crystallites with an average size of approximately 133.8 ± 50.1 nm, as shown in Figure S5a. The increase of the NaOH concentration to 0.7 M (Figure S5b) provoked the formation of nodular-like mesocrystals averaging 221.7 ± 40.5 nm; the change in morphology is likely caused by a 3D self-assembly of the smaller crystals that caused aggregation. This phenomenon is achieved by a secondary dissolution-recrystallisation process occurring at long reaction intervals in the hydrothermal system. In the 1.0 M NaOH solution, the secondary dissolution-recrystallisation process promoted a rapid crystallisation and growth of submicron-sized particles with an average size of 342.4 ± 150.7 nm, exhibiting a plate-like morphology (Figure S5c). The increase of the $OH^-$ content in the hydrothermal media accelerates the dissolution-recrystallisation stage and consequently triggers the preferential crystal growth in the direction perpendicular to the cross section of the plate-shaped particles. These results revealed that the NaOH concentration is critical for achieving a particular $BaCr_2(P_2O_7)_2$ particle crystallisation pathway, which led to different particle morphologies due to a secondary dissolution-recrystallisation process achieved at the longest interval during the hydrothermal synthesis.

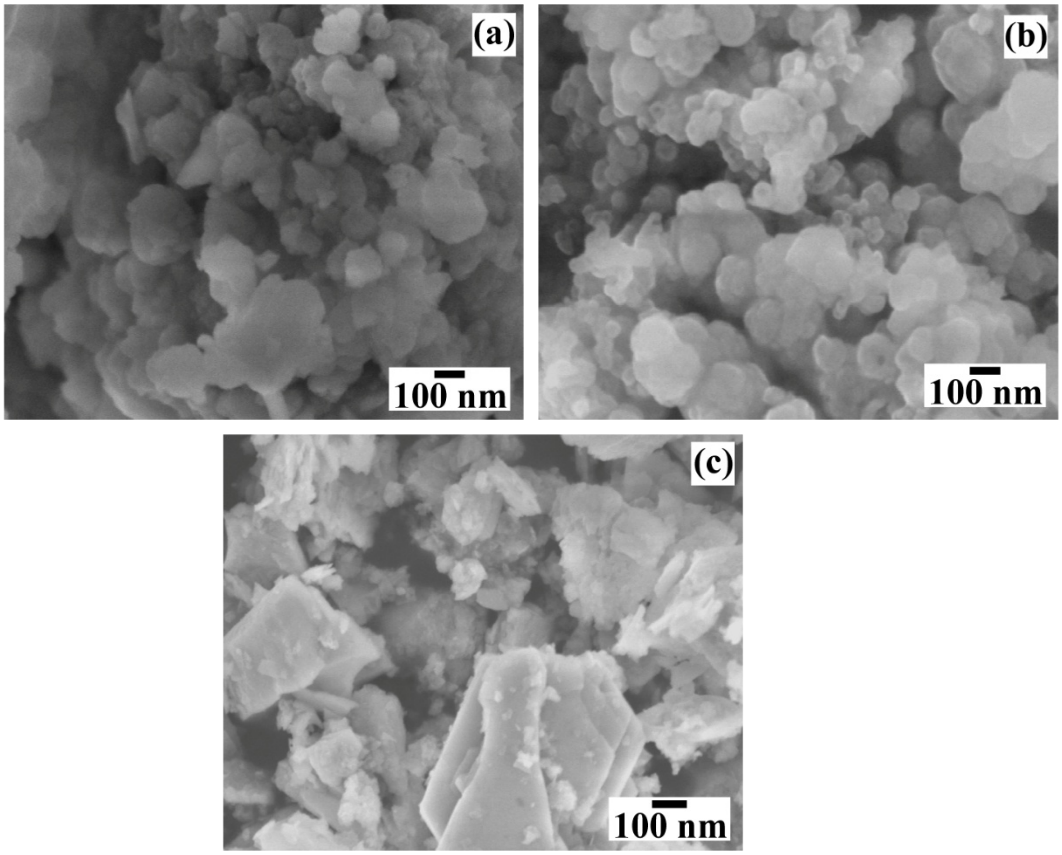


**Figure S5.** FE-SEM micrographs of the $BaCr_2(P_2O_7)_2$ pigments produced under hydrothermal conditions with 50 mmol/dm$^3$ of urea at 240 °C for 48 h in NaOH solutions with concentrations of a) 0.5 M, b) 0.6 M, and c) 1.0 M.

***SF5 Thermal Stability of the Precursor Gel and the Hydrothermally Synthesised $BaCr_2(P_2O_7)_2$ Powders***

The thermal stability of the coprecipitated gel and the dried $BaCr_2(P_2O_7)_2$ powders hydrothermally prepared under experimental conditions at 240 °C for 24 h with 50 mmol/dm$^3$ of urea were carried out by differential scanning calorimetry (DSC–TG) analyses. Typical thermogravimetric and differential calorimetric curves are portrayed in Figure S6. The DSC curve corresponding to the coprecipitated precursor gel ($BaCr_2P_4(OH)_{28}$•$nH_2O$) exhibited an endothermic event at 254 °C, which is attributed to weight loss due to the $OH^-$ ions, which volatilised as water vapour. The total mass loss up to 900 °C was approximately 27.0 wt.%. According to this result, the chemical formula of the coprecipitated gel was $BaCr_2P_4(OH)_{28}$•$1.5H_2O$. Moreover, the differential calorimetric curves of the samples hydrothermally prepared with a 0.5 M NaOH solution exhibited an apparent endothermic event at 417 °C, see Figure S6. This peak is associated with a change in the DSC curve slope because no variation in the weight was observed at this temperature. The DSC curve suggests that other solid–phase transformations do not occur in the temperature range of 417–900 °C; the sample prepared with the highest concentrated NaOH solution of 1.0 M also exhibited similar thermal behaviour compared to the pigment prepared at 0.5 M NaOH.

Furthermore, the weight loss curves of the reaction products synthesised with NaOH concentrations of 0.5 M and 1.0 M revealed an average total weight reduction of approximately 10 wt.% and 13 wt.%, occurring within the temperature range of 100–900 °C, respectively. According to these results, the weight loss contents correspond to 0.55 and 0.72 moles of $H_2O$ vapour, respectively. This weight variation is attributed to the release of $H_4O_4$ ions, which partially substitute oxygen ions within the polyhedral units ($P_2O_7^{4-}$). These

results agree with the intensity differences revealed from the FT–IR spectrum bands of water. Even at the largest content of nominal incorporation of these anions, the synthesised pigment retains structural characteristics similar to those of the pure phase obtained through solid-state reactions, as indicated by the lattice parameters in Table 2

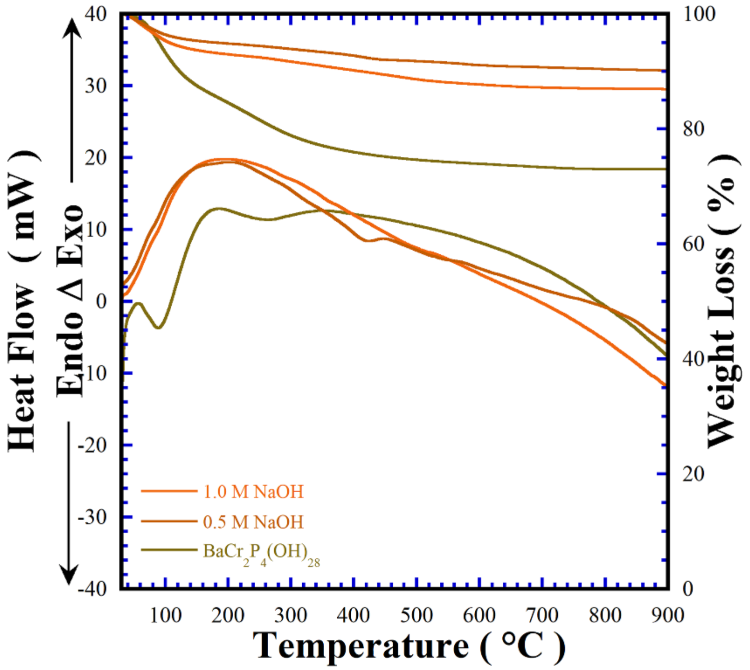


**Figure S6.** Differential scanning calorimetry and thermogravimetry curves of the precursor gel and pyrophosphate powders ($BaCr_2(P_2O_7)_2$) prepared under hydrothermal conditions at 240 °C for 24 h with 50 mmol/dm$^3$ of urea, varying the NaOH solution concentration.

*SF6 Characterisation of the NIR spectral reflectance and the NIR Solar reflectance*

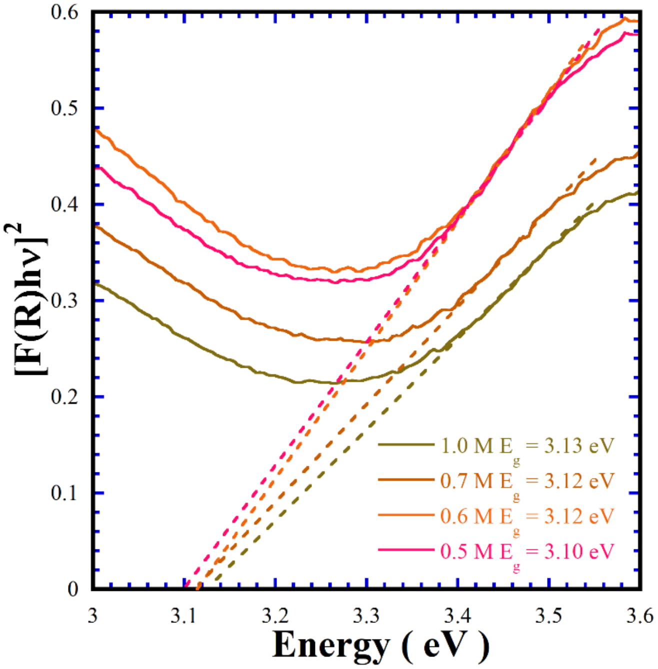


**Figure S7.** Kubelka-Munk curves of $BaCr_2(P_2O_7)_2$ pigments prepared under hydrothermal conditions with 50 mmol/dm$^3$ at 240 °C for 48 h, varying concentration of NaOH solution

Figure S8 portrays the UV-vis NIR reflectance (400–2500 nm) and NIR solar reflectance spectra (800–2500 nm) of $BaCr_2(P_2O_7)_2$ powders synthesised hydrothermally at 240 °C for 24 h with 50 mmol/dm$^3$ of urea varying the NaOH concentration, and the Rutile ($TiO_2$) powder is included for comparison proposes. In the NIR range (800–2500 nm), the $BaCr_2(P_2O_7)_2$ sample prepared with 0.5 M NaOH exhibited an average reflectance of approximately 67.0 %, lower than the $TiO_2$ reference value of 80.0 %. The samples prepared with 1.0 M NaOH exhibited a high reflectance of 72.0 % in the 800–1500 nm region, which is nearly similar to the Rutile reflectance. The marked increase in the pigment's reflectance is attributed to the particle morphology. On the contrary, the powders synthesised with low NaOH concentrations, their reflectance only increased 5.0 %. Thus, these results indicate that the optical pigment's capability depends on the concentration of the hydrothermal alkaline media. This trend is supported by the morphological changes in the particle shape determined by the SEM observations. Additionally, Figure S8b shows the NIR solar reflectance spectra of $BaCr_2(P_2O_7)_2$ samples and the $TiO_2$ standard. The powders synthesised with 0.5 M and 1.0 M NaOH exhibited solar reflectance values of 62.9 % and 67.0 %, respectively, while the rutile reference exhibited a NIR solar reflectance of 79.0 %. The reflectance increases on the samples prepared using a highly concentrated NaOH solution, enhancing the recrystallisation of well-defined plate-like particles, promoting an optimum interaction of the emitted NIR photons on the flat surfaces of these particles. Although the $BaCr_2(P_2O_7)_2$ optical properties are slightly lower than those of $TiO_2$, the solar reflectance values of the $BaCr_2(P_2O_7)_2$ pigments are considered optimum for potential use as energy–efficient cool pigments.

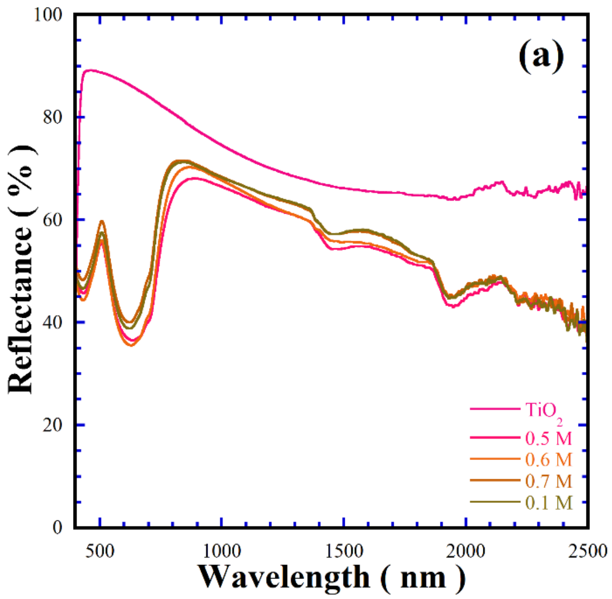


**Figure S8**. a) UV-vis NIR spectral reflectance curves and b) NIR solar reflectance spectra of $BaCr_2(P_2O_7)_2$ pyrophosphate pigments prepared under hydrothermal conditions at 240 °C for 24 h by varying the NaOH concentration and Rutile ($TiO_2$).

Table S2 summarises the literature survey analysis related to the structure, optical, and chromatic features of various green pigments prepared by different methods, including $Cr_2O_3$, $ZnCr_2O_4$, $CoCr_2O_4$, $CoCrAlO_4$ and $Ca_3Cr_2Si_3O_{12}$. The $BaCr_2(P_2O_7)_2$ pigment synthesised by the hydrothermal method is also included for comparison purposes. It deserves to emphasise that the $BaCr_2(P_2O_7)_2$ compound crystallises with a triclinic structure, in comparison to the higher-symmetric structure frameworks of other pigments; namely, trigonal for ($Cr_2O_3$), cubic spinel ($ZnCr_2O_4$, $CoCr_2O_4$, $CoCrAlO_4$) and cubic garnet ($Ca_3Cr_2Si_3O_{12}$). The low crystallographic symmetry of the $BaCr_2(P_2O_7)_2$ lattice promotes anisotropic crystal growth, which enhances diffuse light scattering and contributes to its optical performance [22]. The hydrothermally synthesised $BaCr_2(P_2O_7)_2$ pigment exhibited a maximum solar reflectance of 67.46%, this relative high value indicates that the pigment powder is capable for cold pigment applications, due to it can dissipate more than the 67.0% of the total 52% NIR solar radiation, this behaviour is near to that determined in highly reflective Cr-based green pigments, namely $Ca_3Cr_2Si_3O_{12}$ (90.3%) and $BaCr_2(P_2O_7)_2$ (77.5%) prepared by the hydrothermal and solid-state methods, respectively. Generally, the material's suitability for potential cold pigment applications is correlated to its total solar reflectance, with values above 40.0 % acceptable as cold pigment. The high solar reflectance depicts the material's capability to hinder the pigment heating process triggered by the NIR photons' interaction with the bulk particles; this critical property is relevant for coating applications for friendly environmental building construction and its sustainability, because it decreases the urban island heating phenomena caused by high air conditioning consumption energy.

In addition, the colourimetry data analysis of the green pigments in Table S2 revealed differences in the colour CIEL*a*b* coordinates values; from these data, a correlation of these properties with the pigment's crystal structure is erroneous to establish due to the marked random variation of the data. The microstructural aspects of most of the green pigments in the former literature were not discussed in detail; therefore, a rational correlation in this case cannot be proposed. According to the colour space coordinate data in Table S2, we conclude that the differences in these data strongly depend on the chemical

composition of each pigment in conjunction with the location of the $Cr^{3+}$ species in the crystal structure. It deserves to emphasise that the hydrothermally prepared $BaCr_2(P_2O_7)_2$ pigment exhibits an adequate green tonality, positioning it as a promising alternative to conventional Cr-based green pigments for decorative applications, in addition to the technical abovementioned in this Section.

**Table S2**. Comparison of NIR solar reflectance and colour coordinates for green pigments synthesised by various methods, including the $BaCr_2(P_2O_7)_2$ compound studied.

| Chemical compound | Synthesis method | Crystalline Structure | Solar Reflectance (% R*) | CIELab Coordinates | | | RGB Colour Coordinates | | | Chroma $C_{ab}$* | Colour Hue | Reference |
|---|---|---|---|---|---|---|---|---|---|---|---|---|
| | | | | L* | a* | b* | R | G | B | | | |
| $BaCr_2(P_2O_7)_2$ | Solid-state | Triclinic | 77.50 | 72.5 | -20.5 | 14.7 | 149 | 188 | 150 | 25.23 | | [1] |
| $Ca_3Cr_2Si_3O_{12}$ | Hydrothermal | Cubic | 90.30 | 64.10 | -18.75 | -3.06 | 112 | 165 | 160 | 19.00 | | [18] |
| $Cr_2O_3$ | Solid-state | Trigonal | 65.87 | 38.27 | -12.04 | 8.88 | 75 | 95 | 75 | 14.96 | | [33] |
| $ZnCr_2O_4$ | Sol-gel | Cubic | 59.50 | 71.20 | -6.10 | 6.40 | 168 | 177 | 163 | 8.84 | | [34] |
| $CoCr_2O_4$ | Sol-gel | Cubic | 43.00 | 53.20 | -21.70 | -9.40 | 63 | 138 | 142 | 23.65 | | [34] |
| $CoCrAlO_4$ | Sol-gel | Cubic | 43.70 | 51.10 | -15.4 | -15.60 | 66 | 130 | 148 | 21.92 | | [34] |
| $BaCr_2(P_2O7)_2$ | Hydrothermal | Triclinic | 67.46 | 66.41 | -15.48 | -2.74 | 126 | 170 | 166 | 15.72 | | This work |

## References


1. Tao, Z.; Zhang, W.; Huang, Y.; Wei, D.; Jin, S.H. A novel pyrophosphate $BaCr_2(P_2O_7)_2$ as green pigment with high NIR solar reflectance and durable chemical stability. *Solid State Sci.* **2014**, *34*, 78–84.

18. Rendón–Angeles, J.C.; Matamoros–Veloza, Z.; Rodríguez–Galicia, J.L.; Seong, G.; Yanagisawa, K.; Tamayo, A.; Rubio, J.; Anaya–Chavira, L.A. One–pot hydrothermal synthesis of Victoria green ($Ca_3Cr_2Si_3O_{12}$) nanoparticles in alkaline fluids and its colour hue characterization. *Nanomaterials.* **2021**, *11*, 521.

22. Bandara, P.C.; Peña-Bahamonde, J.; Rodrigues, D.F. Redox mechanisms of conversion of Cr(VI) to Cr(III) by graphene oxide-polymer composite. *Sci. Rep.* **2020**, *10*, 9237.

33. Oka, R.; Masui, T. Synthesis and characterization of black pigments based on calcium manganese oxides for high near–infrared (NIR) reflectance. *RSC. Adv.* **2016**, *6*, 90952–90957.

34. Yu, J.; Jiang, F.; Liu, J.; Wang, T.; Zhang, X.; Zhang, X.; Zhang, Q.; Zhang, R.; Wu, Q.; Hu, Q.; et al. Coloring and near-infrared reflection performance of low temperature synthesized novel (Cr, V)-$ZrSiO_4$ jewel green pigments. *Ceram. Int.* **2023**, *49*, 38602–38613.
